# Beliefs About the Development of Mental Life

**DOI:** 10.1162/opmi_a_00200

**Published:** 2025-04-22

**Authors:** Kara Weisman, Lucy S. King, Kathryn Humphreys

**Affiliations:** Department of Psychology, University of California, Riverside; Department of Psychology and Human Development, Vanderbilt University

**Keywords:** child development, cognitive development, emotional development, folk theories, mind perception, parenting, social development

## Abstract

Caregiving relationships with infants and children are among the most common and most complex human social interactions. Adults’ perceptions of children’s mental capacities have important consequences for the well-being of children in their care—particularly in the first few years of life, when children’s communication skills are limited and caregivers must infer children’s rapidly developing thoughts, feelings, and needs. In a series of studies, we assessed how US adults conceptualize the development of the human mind over the first five years of life. Exploratory factor analysis identified four core capacities that anchored participants’ representations of the developing human mind: *bodily sensation* (e.g., hunger, pain), *negative affect* (e.g., distress, frustration), *social connection* (e.g., love, learning from others), and *cognition and control* (e.g., planning, self-control). Participants believed that these capacities were present to different degrees at birth, followed different developmental trajectories, and were driven by different developmental mechanisms, such as biological “preprogramming,” physical maturation, passive observation, and social learning. The current studies shed light on this fascinating and understudied aspect of “mind perception” among US adults, in turn highlighting possibilities for theory-based interventions to encourage developmentally appropriate parenting behaviors.

## INTRODUCTION

Frustration, compassion, worry, humor, imagination, love—such experiences are fundamental parts of human life, but few people would assert that these capacities are fully developed at birth. In any given interaction with a child, adults come face to face with a being whose experience of the world is like their own, but different; whose mind is capable of some of the thoughts and feelings that an adult’s mind is, but perhaps not all of them. When an adult interacts with the same child over time, they witness innumerable changes in that child’s mental life: A sleepy newborn gradually becomes a child who experiences complex emotions and original ideas and develops personal memories and intricate plans. This familiar but remarkable transformation requires adults to make inferences about a given child’s developing mental capacities in order to interpret that child’s behaviors and respond appropriately.

Indeed, caring for a young child involves making countless decisions under uncertainty: Why is my child crying, smiling, shouting, or biting? What do they want, and what do they need? Can they understand what I am saying? Do they love me? As in other domains of reasoning (Gerstenberg & Tenenbaum, 2017; Gopnik & Wellman, 1994; Wellman & Gelman, 1992), people likely draw on folk beliefs and intuitive theories about how the mind develops to predict, explain, and respond to children’s behavior and development, with consequences for the well-being of children in their care.

These folk beliefs and intuitive theories are, to date, poorly understood by social scientists. A handful of recent studies have examined the extent to which cognitive abilities, knowledge, and emotions are thought to be innate vs. learned, with special attention to comparing laypeople’s beliefs about innateness to scientific evidence for or against innateness and universality (Berent et al., 2019, 2020; Wang & Feigenson, 2019; see Berent, 2021, for discussion). These studies offer a fascinating glimpse of potential gaps, misconceptions, and biases in laypeople’s beliefs about the human mind, but they leave open many questions about the nature of the folk beliefs and intuitive theories themselves. What is the cognitive architecture underlying laypeople’s beliefs about the origins of the human mind? How do laypeople “carve up” the various sensations, experiences, and abilities that make up human mental life, and—most crucially from the perspective of caregiving—how do laypeople understand changes in mental life across early development?

In this paper, we examine how US adults conceptualize the developing minds of infants and children by drawing on techniques developed in studies of mind perception, in which researchers use large datasets and modern statistical methods to reveal conceptual representations of “the mind,” broadly construed. In a groundbreaking study, Gray et al. asked participants to compare the mental capacities of a range of humans, animals, technologies, and other entities, and argued that participants’ judgments revealed a fundamental distinction between “agency” (e.g., capacities for self-control, moral reasoning, memory, and planning) and “experience” (e.g., capacities for hunger, fear, pain, and pleasure, Gray et al., 2007). More recently, Weisman et al. used a similar empirical approach to argue that people’s conceptions of mental life are anchored by three fundamental components: “body” (physiological sensations such as hunger and pain), “heart” (social-emotional abilities such as embarrassment and pride), and “mind” (perceptual-cognitive capacities such as vision and memory, Weisman et al., 2017b; see Malle, 2019, for a similar three-factor solution). Such findings have inspired subsequent work on topics as wide-ranging as moral reasoning, dehumanization and stereotyping, beliefs about the afterlife, and human-robot interactions.

However, the burgeoning field of mind perception has largely neglected the question of how the human mind changes over development. In many studies, infants and children have simply not been included as targets for mental capacity attributions; instead, participants have been asked to reason about human adults alongside a range of non-human entities, including other animals, technologies, and supernatural beings. Even studies that have probed participants’ mental capacity attributions to infants and children have generally treated them as beings distinct from adults, rather than as snapshots of a single mind as it emerges and changes over time. For example, Gray et al.’s early work on mind perception suggested that a five-month-old infant is an entity that is generally considered to have experience but not agency—in contrast to, say, a five-year-old child (considered to have experience as well as limited agency), a human adult (considered to have maximal experience and agency), or a robot (considered to have agency but not experience, Gray et al., 2007). Likewise, Weisman et al.’s studies characterized infants and young children—along with, e.g., chimpanzees, elephants, and dolphins—as entities that are perceived to be fully capable of physiological sensations but more limited in their perceptual-cognitive capacities and social-emotional abilities; this is in contrast to human adults, who are perceived to be fully capable across these domains (Weisman et al., 2017b). Although such findings can be reinterpreted as providing preliminary evidence that people consider the minds of infants and children to differ from the minds of adults, they do not address how people conceptualize the development of the mind in early life.

Yet infants and young children are far from unusual social partners. Caregiving relationships are at the core of human existence; for many people, infants and young children are among the most frequent and highly valued minds that we encounter. In the current studies we set out to shed new light on this important and understudied aspect of mind perception: how laypeople reason about the development of human mental life. In three studies, we assessed US adults’ folk beliefs about children’s developing abilities for physiological sensation, perception, cognition, emotion, self-regulation, social interaction, and other aspects of “mental life,” broadly construed. We focused on development between birth and age five years—a time of particularly dramatic maturation in perceptual acuity, emotional experience, cognitive ability, executive function, social engagement, and many other domains.

Methods, inclusion/exclusion criteria, and analyses marked as “preregistered” were preregistered on the Open Science Framework (OSF) website (Study 1: https://osf.io/e6ajh; Study 2: https://osf.io/j72dg; Study 3: https://osf.io/xh8ce). All studies were approved by the Stanford University Research Compliance Office (Human Subjects IRB). For extended descriptions of methods and results, see Supplemental Materials. Note: scripts for all analyses, figures, and tables are available in the .Rmd file that generated the fully reproducible Supplemental Materials document (supplement-main.Rmd), which can be found in the following GitHub repository: https://github.com/kgweisman/baby_mental_life_ms.

## STUDY 1

The first question we sought to address in this line of work was: What do US adults perceive to be the fundamental components of mental life, as they pertain to a developing human child? To this end, we began with an expansive exploration of US adults’ attributions of mental life to infants and young children in general; this laid the foundation for Studies 2–3, which we designed to assess the perceived developmental trajectories of these attributions across specific target ages.

Drawing inspiration from previous work on mind perception (Gray et al., 2007; Weisman et al., 2017a, 2017b, 2018) as well as standard assessments of infant temperament and behavior (Putnam et al., 2006; Rothbart, 1978), we included a wide range of 60 capacities in this initial exploration, from basic physiological sensations, to capacities for perception, cognition, and emotion, to abilities for self-regulation and social interaction. We asked participants to assess these capacities at three different ages (at birth, 9 months, and 5 years), with the goal of characterizing the conceptual structure underlying participants’ reasoning about the development of mental life.

We designed Study 1 to explore the correlational structure of participants’ responses, as a window into the underlying conceptual structure that might support their reasoning about the development of mental life. Our primary analysis was a preregistered exploratory factor analysis (EFA) of participants’ capacity ratings. This analysis allowed us to examine which capacities tend to “hang together” in participants’ assessments of the mental lives of infants and young children. For example, when a participant indicated that newborns are highly capable of feeling scared, what other capacities did they tend to attribute to newborns? Following previous work on mind perception, we argue that the suites of capacities revealed by applying dimensionality reduction (in our case, EFA) to this covariance structure offer a meaningful approximation of the latent conceptual structure underlying participants’ reasoning about the developing human mind.

### Methods

#### Participants.

301 US adults participated via Amazon Mechanical Turk (MTurk) in July–August 2018. Participants ranged in age from 19–45 years (*M* = 31.37 years, *sd* = 5.75 years) and included more men (59%) than women (41%; < 1% of participants identified as some other gender or declined to disclose). Participants predominantly identified as White (66%; < 15% identified as any other race/ethnicity, identified as more than one race/ethnicity, or declined to disclose). 51% of participants had obtained at least a Bachelor’s degree. 44% of participants indicated that they were parents.

#### Materials and Procedure.

Participants completed three trials in which they assessed the mental lives of children at three target ages: birth, 9 months, and 5 years. On each trial, they were shown two representative photographs of children at the target age (labeled “newborns,” “9-month-olds,” or “5-year-olds”), and asked to answer the following question for 60 capacities: “To what extent is a [newborn/9-month-old/5-year-old] capable of [this capacity]?” See Supplemental Materials for details about the selection of these photographs. These photographs (and all materials used in these studies) are available in the following OSF repository: https://osf.io/xrznd. Participants responded on a sliding scale from 0 (labeled “not at all capable”) to 100 (“completely capable”).

**Figure 1.**
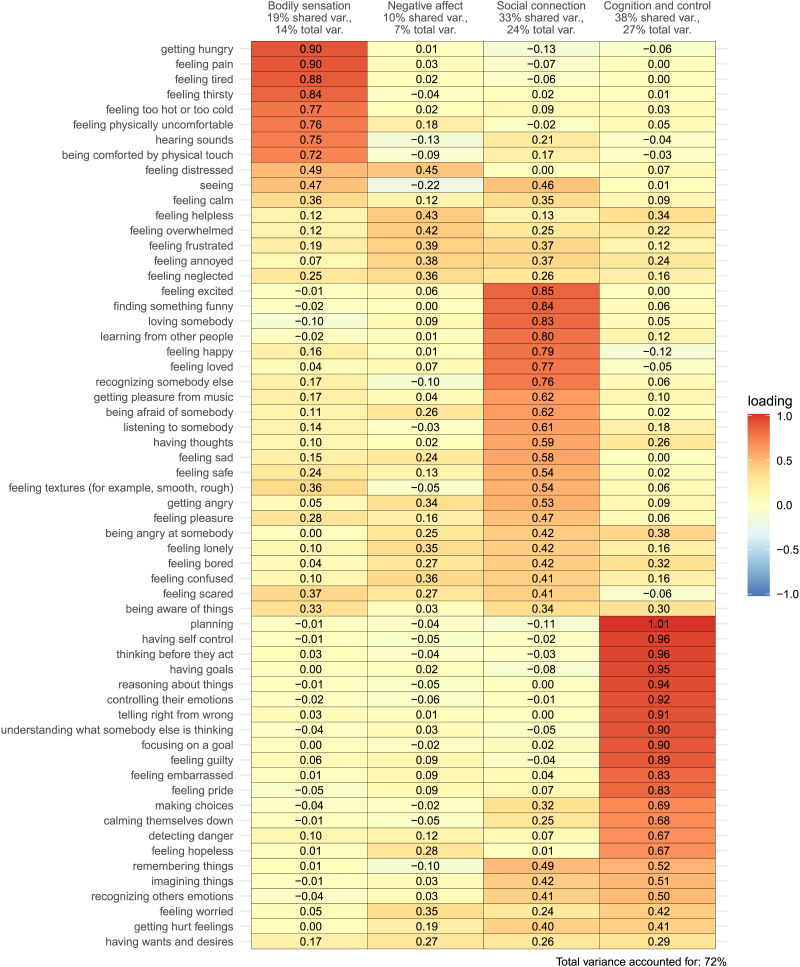
Factor loadings from an exploratory factor analysis of US adults’ capacity attributions to newborns, 9-month-old infants, and 5-year-old children in Study 1.

The 60 capacities included in Study 1 were drawn from several areas of previous research. Our goals in selecting items were to: (1) balance the representation of the conceptual organization underlying mental capacity attributions in general, as identified in previous work (Gray et al., 2007; Malle, 2019; Weisman et al., 2017a, 2017b, 2018); (2) add items relevant to early development, as identified by existing measures of temperament and behavior in early life (Putnam et al., 2006; Rothbart, 1978); and (3) assess the broadest range of mental capacities within financial and participant burden constraints, as identified by extensive discussions among the authors drawing on our own research and clinical experience with infants and young children. This process yielded a list of 60 capacities; see Figure 1, and see Supplemental Materials for an item-by-item comparison of the capacities used here to those used in previous studies of mind perception. Capacities were presented in a random order.

In planning this series of studies, we selected 13 target ages based on the developmental milestone ages identified by the CDC (Centers for Disease Control and Prevention, 2021) with the addition of the American Academy of Pediatrics’ recommendations for pediatrician visits before 2 months (American Academy of Pediatrics, 2018): birth, 4 days, 1 month, 2 months, 4 months, 6 months, 9 months, 12 months, 18 months, 2 years, 3 years, 4 years, and 5 years. We limited Study 1 to just the youngest, median, and oldest target ages—presented to participants as “newborns,” “9-month-olds” and “5-year-olds”—in order to maximize the number of capacities included in the study without over-burdening participants.

Target ages were presented in chronological order, with participants first assessing all 60 capacities for newborns, then 9-month-old infants, and finally 5-year-old children. In this and all studies, we opted for this fixed-order design because our priority was to assess beliefs about the developmental trajectories of capacities, rather than beliefs about capacities at a specific point in development. The fixed order of target ages was intended to ensure that questions about developmental trajectories “flowed” in a coherent way (Krosnick & Presser, 2009).

### Results

Following recent work on mind perception (Weisman et al., 2021; Weisman, Dweck, & Markman, 2007), our goal in this analysis was to derive a set of latent constructs that together give rise to people’s intuitions about the development of human mental life between 0–5 years. We did not set out to sort each of the 60 capacities into discrete, non-overlapping categories of capacities. Instead, we understood each of these specific capacities to reflect a different combination of the same underlying components (much as specific colors of paint can be understood as different combinations of cyan, magenta, and yellow). This theoretical perspective motivated our choice of exploratory factor analysis as our dimensionality reduction technique (rather than, e.g., principal components analysis or cluster analysis); our choice of labels for each factor, in which we have attempted to describe what might be understood by participants as core components of the developing human mind (rather than attempting to label a category); and our interpretation of factor loadings, which focuses not only on capacities that load strongly on only one factor but also on capacities that cross-load on multiple factors. See Supplemental Materials for more details about this analysis, including our decision to treat participants’ ratings of each of the three target ages as if they were a distinct source of data, and solutions employing orthogonal (varimax) rotation rather than the oblique (oblimin) transformations presented here.

A 4-factor solution (as suggested by parallel analysis) accounted for 72% of the total variance in participants’ capacity attributions, suggesting that four latent constructs can account for a substantial amount of the variability across target ages and across individual participants in perceptions of infants’ and young children’s mental lives. See Figure 1 for all factor loadings after oblique transformation, and see Supplemental Materials for a scatterplot matrix (“pairs plot”) and related plots visualizing the relationships among factor loadings across these four factors and the distributions of factor loadings across items (Figures S1–S7); as well as factor loading for the 8-factor solution suggested by minimizing BIC, and the 2-factor solution suggested by implementing the factor retention criteria employed in Weisman et al. (2017).

The first factor corresponded primarily to capacities related to thinking, reasoning, agency, and executive function—a suite of capacities that we interpreted as reflecting a higher-order capacity for *cognition and control*. It was the dominant factor for such items as “planning,” “having self-control,” “thinking before they act,” “having goals,” “reasoning about things,” “controlling their emotions,” “telling right from wrong,” “understanding what somebody else is thinking,” “focusing on a goal,” “feeling guilty,” “feeling embarrassed,” “feeling pride,” “making choices,” “calming themselves down,” “detecting danger,” and “feeling hopeless.” (For this and the following factors, we list all of the items with loadings ≥0.60 in descending order by factor loading; see Figure 1 for the full set of loadings.) This factor accounted for 38% of the shared variance in the rotated 4-factor solution and 27% of the total variance in participants’ capacity attributions.

The second factor corresponded primarily to what we would describe as social abilities and emotional experiences, particularly those with positive valence—a suite of capacities that we that we interpreted as reflecting a higher-order capacity for *social connection*. It was the dominant factor for such items as “feeling excited”, “finding something funny”, “loving somebody”, “learning from other people”, “feeling happy”, “feeling loved”, “recognizing somebody else”, “getting pleasure from music”, “being afraid of somebody,” and “listening to somebody.” Although some of these capacities do not require the presence of another person, we use the term “social connection” both to reflect the fact that many of the highest-loading items do highlight overtly “social” capacities (e.g., humor, love, learning from others, recognizing others, fearing others, listening to others), and because we interpret this factor as indexing participants’ sense of the infant awakening into the human social and cultural world. This factor accounted for 33% of the shared variance in the rotated 4-factor solution and 24% of the total variance in participants’ capacity attributions.

The third factor corresponded primarily to physiological sensations related to biological needs—a suite of capacities that we interpreted as reflecting a higher-order capacity for *bodily sensation*. It was the dominant factor for such items as “getting hungry”, “feeling pain”, “feeling tired”, “feeling thirsty”, “feeling too hot or too cold”, “feeling physically uncomfortable”, “hearing sounds”, and “being comforted by physical touch”, and accounted for 19% of the shared variance in the rotated 4-factor solution and 14% of the total variance in participants’ capacity attributions.

Finally, the fourth factor corresponded to negatively-valenced sensations and emotions—a suite of capacities that we interpreted as reflecting a higher-order capacity for *negative affect*. Although there were no items that loaded more strongly than 0.45 on this factor, this factor was the dominant factor for the items “feeling helpless”, “feeling overwhelmed”, “feeling frustrated”, “feeling annoyed”, and “feeling neglected,” and the items “feeling distressed”, “feeling confused”, “feeling worried”, and “feeling lonely” also loaded relatively strongly on this factor (albeit slightly more strongly on other factors). This factor accounted for 10% of the shared variance in the rotated 4-factor solution and 7% of the total variance in participants’ capacity attributions.

How robust are these findings? The factors we have called *bodily sensation, social connection*, and *cognition and control* each had strong factor loadings (≥0.60) for many individual items (*n* = 8–16) and each accounted for a substantial amount of the total variance in participants’ capacity attributions (14–27%), indicating three robustly distinct and interpretable factors. In contrast, the fourth factor—*negative affect*—had more moderate loadings even for its strongest-loading items (all loadings < 0.46) and accounted for only 7% of the total variance. Nonetheless, we opted to retain this factor in subsequent studies because we considered the general domain of *negative affect* to be of particular theoretical and clinical interest. An individual’s propensity to negative affect in infancy and early childhood is a salient individual difference trait associated with risk for internalizing disorders (Gartstein et al., 2012). Beyond this, positive and negatively valenced emotionality emerge at different developmental timescales: Signs of negative affect are present from birth (e.g., crying shortly following delivery), whereas signs of positive affect (e.g., social smiling, laughing) are not present for several weeks (Humphreys et al., 2015), supporting both the relevance and distinctiveness of negative emotional expression in early life.

It is worth noting that a handful of items loaded relatively strongly on two factors (loadings ≥0.40), suggesting that these factors are not entirely distinct. In our view, these cross-loadings were generally sensible. The item “feeling distressed”, for instance, loaded roughly equally strongly on both *bodily sensation* and *negative affect*—perhaps reflecting the idea that “distress” might be experienced both as a physical sensation (similar to pain) and an affective state (similar to fear or frustration). Likewise, “seeing” loaded equally strongly on both *bodily sensation* and *social connection*—perhaps reflecting an understanding of vision as both a basic sensory ability (closely tied to a particular body part) and a critical part of many social interactions (especially stereotypical interactions with infants, which often hinge on making eye contact, imitating facial expressions, playing peekaboo, etc.).

As a check on our choice of labels for these factors, we turned to OpenAI’s Generative Pre-trained Transformer (ChatGPT, version 4o, OpenAI, 2024), an autoregressive transformer-based language prediction model trained on an enormous corpus of English-language text drawn primarily from “crawls” of the Internet as well as English-language novels and the open-source encyclopedia Wikipedia. Like other generative language models, ChatGPT was developed under the mandate of predicting the continuation of natural language sequences: Most concretely, then, its “responses” should be understood to reflect a sequence of words that is likely to follow the prompt it has been provided. In a more abstract sense, however, the content of these responses could be understood to represent the “common knowledge” embedded in the training data (for extended arguments relevant to this proposal, see Bhatia & Richie, 2024; Lake & Murphy, 2023; Petroni et al., 2019). For example, if prompted with the questions “What do you get when you mix red and yellow?” or “What’s a good name for a vegetarian restaurant?,” ChatGPT will generate text like “When you mix red and yellow, you get orange” or “The Green Plate” (respectively). In continuing the language sequence, the model also provides novel content that reflects actual human knowledge, biases, associations, and intuitions.

We provided a variety of prompts to the model to assess its “intuitions” about how to label the four categories of mental capacities suggested by the factor solution just described. One representative prompt began, “You’re a statistician and a psychologist, and I’d like your help interpreting my exploratory factor analysis results,” followed by the EFA output taken directly from our analysis in R (containing the full list of factor loadings, as well as other summary characteristics of the EFA solution). The response generated by ChatGPT included comments on factor loadings and structure, factor statistics, model fit, communalities and item complexity, factor score adequacy, key takeaways, and next steps. Under factor loadings, it read, “Factor 1 (MR1) includes items related to self-regulation and cognitive control, like ‘controlling their emotions’ and ‘planning.’ Factor 2 (MR2) encompasses physical sensations and discomfort, such as ‘feeling physically uncomfortable’ and ‘getting hungry.’ Factor 3 (MR3) relates to emotional experiences and social connection, with items like ‘feeling happy’ and ‘loving somebody.’ Factor 4 (MR4) is smaller but captures emotional reactivity and distress, such as ‘feeling distressed’ and ‘feeling overwhelmed.’ ” Under key takeaways, it read, “Factor labels could be: MR1: Self-Regulation and Cognitive Control; MR2: Physical Sensations and Discomfort; MR3: Emotional Experiences and Social Connections; MR4: Emotional Reactivity and Distress.” (See https://chatgpt.com/share/677d3926-b284-8006-a37a-ea7543a7a169 for the full text of these prompts and ChatGPT’s responses.) The similarity between our original labels and those generated by ChatGPT provides some evidence that the labels used are consistent with common knowledge and intuitions reflected in the vast swath of English language text that constituted the training data for this model, and by extension with the common knowledge and intuitions shared by many lay people in the English-speaking world. Nonetheless, we caution the reader to treat these labels as interpretations of empirical results, rather than empirical results themselves.

What does it mean that these four factors emerged? At a most basic level, our results reflect sets of intercorrelated capacities: When a participant judged that a child of a certain age was highly capable of planning, they also tended to judge that that child was capable of having self control, thinking before they act, and the other capacities with strong factor loadings on the *cognition and control* factor; and likewise for the suites of capacities that define the other factors revealed by EFA. As described earlier, we take these factors to reflect latent constructs—fundamental components of mental life relevant to reasoning about human children. Moreover, because each participant was asked to reason not just about some class of target characters (e.g., “chimpanzees,” “robots”), but about the human child over development, we further propose that part of what contributed to the differentiation of these four factors was participants’ perceptions of development: the extent to which different capacities are present at birth, the rate at which different capacities develop, and the mechanisms that drive this development. In Studies 2 and 3, we explore these possibilities in greater depth. (See also Supplemental Materials for the perceived developmental trajectories surfaced by Study 1.)

## STUDY 2

In Study 2, we replicated the conceptual structure identified by Study 1 and then used it to chart how different aspects of mental life are perceived to change over development: What kinds of abilities do US adults believe are present at birth, and to what degree? To what extent are capacities for *bodily sensation, negative affect, social connection*, and *cognition and control* perceived to change over childhood, and what is the shape of these perceived developmental trajectories?

The design of Study 2 was nearly identical to Study 1, except that instead of assessing 60 capacities for 3 target ages, each participant assessed 20 capacities for 13 target ages: birth, 4 days, 1 month, 2 months, 4 months, 6 months, 9 months, 12 months, 18 months, 2 years, 3 years, 4 years, and 5 years (see Methods). This design allowed us to chart perceived developmental trajectories with a high degree of precision without undue burden to participants.

### Methods

#### Participants.

304 US adults participated via MTurk in August 2018. Eligibility requirements were identical to Study 1. Participants ranged in age from 19–45 years (*M* = 32.14 years, *sd* = 6.32 years) and included roughly equal numbers of men (51%) and women (49%). Participants predominantly identified as White (75%; < 11% identified as any other race/ethnicity, identified as more than one race/ethnicity, or declined to disclose). 50% of participants had obtained at least a Bachelor’s degree. 41% of participants indicated that they were parents.

#### Materials and Procedure.

Participants completed 13 trials in which they assessed the mental lives of children at 13 target ages: birth, 4 days, 1 month, 2 months, 4 months, 6 months, 9 months, 12 months, 18 months, 2 years, 3 years, 4 years, and 5 years. On each trial, they were shown two representative photographs of children at the target age, and asked to answer the following question for 20 capacities: “To what extent is a [newborn/4-day-old/etc.] capable of [this capacity]?” As in Study 1, participants responded on a sliding scale from 0 (labeled “not at all capable”) to 100 (“completely capable”).

In order to ask participants to assess a more fine-grained array of target ages without undue participant burden, we limited our list of capacities to 20 of the 60 capacities used in Study 1. To identify this list, we drew on the results of Study 1, selecting 5 items for each of the 4 factors identified there. For each factor, we aimed to select items that loaded strongly on that factor, did not cross-load strongly on other factors, were sufficiently distinguishable from each other in meaning, and captured our qualitative understanding of the latent construct that each factor corresponded to. To represent *bodily sensation*, we chose the items “getting hungry”, “feeling pain”, “feeling tired”, “feeling physically uncomfortable”, and “hearing sounds”. To represent *negative affect*, we chose the items “feeling distressed”, “feeling lonely”, “feeling frustrated”, “feeling helpless”, and “feeling overwhelmed”. To represent *social connection*, we chose the items “feeling excited”, “finding something funny”, “loving somebody”, “learning from other people”, and “feeling happy”. Finally, to represent *cognition and control*, we chose the items “planning”, “having self-control”, “reasoning about things”, “controlling their emotions”, and “telling right from wrong”. Capacities were presented in a random order.

The target ages for this study were based on the developmental milestone ages described in Study 1. As in Study 1, target ages were presented in chronological order, with participants first assessing all 20 capacities for newborns, then 4-day-old infants, and so forth, with the primary goal of assessing beliefs about changes in these capacities over development.

### Results

A preregistered EFA yielded four factors very similar to the four factors from Study 1, with all capacity items loading strongly on the factors that they were selected to represent and less strongly on other factors. (See Supplemental Materials for this 4-factor solution, which was suggested by both parallel analysis and Weisman et al.’s [2017] factor retention criteria, as well as the 8-factor solution suggested by minimizing BIC). Minimally, this suggests that our selection of mental capacities for this study captured the essential meaning of the four factors revealed in Study 1, including the factor we have labeled *negative affect*: In Study 2, this factor accounted for fully 25% of the shared variance and had strong factor loadings (≥0.60) for all 5 of the items selected to represent it, none of which loaded strongly on other factors. Beyond this, we consider these results to be a somewhat independent conceptual replication of Study 1 results and validation of our interpretation of these factors. After all, given that Study 2 featured only 20 of the 60 capacities featured in Study 1, it certainly could have been the case that this analysis would suggest retaining fewer factors (as often occurs with fewer variables). Conversely, given that Study 2 featured 13 within-subjects observations for each capacity compared to the 3 in Study 1, this analysis could have surfaced more than four factors (as often occurs with more observations). Finally, if we had misinterpreted the fundamental semantic content that distinguished the factors in Study 1 and selected inappropriate capacities to represent these factors, the Study 2 analysis could have identified more capacities with cross-loadings. Instead, the results of this analysis offer some validation of the four factors we have called *bodily sensation, negative affect, social connection*, and *cognition and control*.

**Figure 2.**
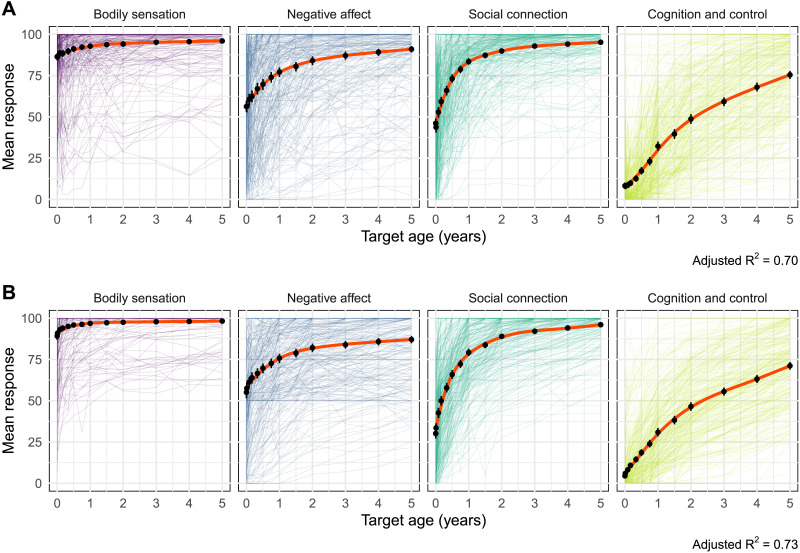
Perceived developmental trajectories for four domains of mental life among US adults (Studies 2–3). Lighter lines represent individual participants’ responses, black points correspond to mean responses across the sample, error bars are bootstrapped 95% confidence intervals, and thick red lines are predictions from our multilevel generalized additive models. In Study 2 (Panel A), participants assessed 5 capacities within each domain, and assessed all capacities for a given target age before moving on to the next target age. In Study 3 (Panel B), participants assessed 2 capacities within each domain, and assessed a single capacity for all target ages before moving on to the next capacity.

Regressing participants’ item-level responses onto target age, domain (assigned a priori; see preregistration), and interactions between them via a multilevel generalized additive model (using a binomial distribution and the logit link function) confirmed both of our preregistered hypotheses: On average, participants rated older children as more capable, but perceptions of the development of children’s capacities differed dramatically across domains. Modeling the effects of target age via smoothing terms allowed us to reconstruct the shape of these perceived developmental trajectories from the bottom up (see Figure 2), without specifying in advance the shape of the curve.

On one extreme, participants attributed very little capacity for *cognition and control* to newborns (median score for newborns: 1.00 out of 100 points, *M* = 8.24, *sd* = 18.61), but reported that such capacities increase dramatically and non-linearly over the target age range–that is, including a smoothing term for target age (analogous to a slope in a linear regression) significantly reduced the deviance of the model (*χ*^2^ = 4537.42, *p* < 0.001); and the fitted smooth had moderate non-linear complexity (estimated degrees of freedom *edf* = 5.79). A visual inspection of Figure 2 (Panel A, rightmost box) suggests that participants perceived a long and fairly steady developmental trajectory across the full target age range included in this study (birth to 5 years).

Compared to these attributions of *cognition and control*, participants attributed substantially greater capacities to newborns in the domain that we have labeled *social connection* (median score for newborns: 45.00 out of 100 points, *M* = 46.06, *sd* = 37.30; increase in log-odds relative to *cognition and control: β* = 2.79, *p* < 0.001), but still perceived dramatic non-linear growth in this domain over the target age range (*χ*^2^ = 3196.67, *p* < 0.001, *edf* = 6.93), characterized by rapid development over the child’s first year of life (see Figure 2, Panel A, third box from left).

In the domain of *negative affect*, participants attributed even greater capacities to newborns (median score for newborns: 63.50 out of 100 points, *M* = 56.24, *sd* = 38.04; increase in log-odds relative to *cognition and control: β* = 2.55, *p* < 0.001) and smaller increases over the target age range (*χ*^2^ = 3196.67, *p* < 0.001, *edf* = 6.93), concentrated primarily between birth and the age of 2 years (see Figure 2, Panel A, second box from left).

In the domain of *bodily sensation*, participants perceived hardly any growth over the target age range; instead, participants attributed nearly maximal capacity to newborns (median score for newborns: 100.00 out of 100 points, *M* = 86.42, *sd* = 24.28; increase in log-odds relative to *cognition and control: β* = 4.15, *p* < 0.001) and reported the smallest increase increase in capacities over the target age range (*χ*^2^ = 281.51, *p* < 0.001, *edf* = 3.86). What growth they did perceive occurred in the first year (see Figure 2, Panel A, leftmost box).

We speculate that these differences in perceptions of growth in children’s capacities emerge from intuitive theories of development that specify different mechanisms or drivers of development for different domains. For example, if participants believe that development in the domain of *bodily sensation* is primarily driven by innate biological forces, they might assume that most of this development is complete before birth, leaving little room for change over the target ages assessed. (See Berent et al., 2019, for empirical evidence in line with this speculation.) In contrast, in a domain where participants believe development depends on observation, exploration, or social learning, they might think that children need time in the world to change and grow, yielding lower estimates of capacities at birth and slower estimates of rates of change; or, in a domain where participants believe that development occurs through explicitly teaching the child something, they might perceive that development proceeds nonlinearly, such that large changes occur following specific milestones (e.g., beginning preschool). We explore these possibilities in Study 3.

## STUDY 3

Study 3 was designed with two goals in mind. First, we aimed to provide a more direct assessment of the differences in perceived developmental trajectories surfaced by Study 2. In Study 2, participants focused on one target age at a time, assessing a variety of capacities for that age before proceeding to the next target age (from youngest to oldest). This design was intended to encourage participants to focus primarily on providing a holistic assessment of mental life at a given target age; the trajectories connecting target ages were likely not the participants’ primary concern in the moment of responding. In Study 3, we flipped the design to focus participants’ attention on development more explicitly: Participants were asked to provide judgments of a single capacity for the full range of target ages (arranged from youngest to oldest) on the same “trial,” before proceeding to the next capacity. In addition to signaling more strongly to participants that we were interested in their perceptions of development, this approach gave participants the opportunity to view and adjust all of their responses for a given target capacity simultaneously, charting out their perceptions of the full developmental trajectory from birth to age 5 years.

Second, we aimed to probe the intuitive theories that might underlie the differentiation of the four factors surfaced by Studies 1–2 and the perceived developmental trajectories surfaced by Study 2 (and the current study).

### Methods

#### Participants.

301 US adults participated via MTurk in April 2019. Eligibility requirements were identical to Studies 1–2. Participants ranged in age from 18–45 years (*M* = 31.67 years, *sd* = 5.78 years) and included roughly equal numbers of men (53%) and women (47%). Participants predominantly identified as White (67%; < 12% identified as any other race/ethnicity, identified as more than one race/ethnicity, or declined to disclose). 53% of participants had obtained at least a Bachelor’s degree. 34% of participants indicated that they were parents.

#### Materials and Procedure.

Participants completed eight trials, in which they assessed each of the eight capacities in turn; the order of capacities was randomized across participants. On each trial, participant assessed children at all 13 target ages used in Study 2 in a fixed order from youngest to oldest. Each age was presented along with a representative pair of photographs (also used in Studies 1 and 2), and participants were asked to answer the following question: “To what extent is a [newborn/4-day-old/1-month-old/etc.] capable of [this capacity]?” Participants responded on a sliding scale from 0 (labeled “not at all capable”) to 100 (“completely capable”).

On each trial, participants completed a set of questions about the possible mechanisms that drive development for the capacity in question. First, they were asked, “In your opinion, how important is each of the following factors in the development of children’s capacity for [capacity]?” They provided independent ratings on a Likert-type scale from 0 (labeled “not at all important”) to 6 (“extremely important”), for the following 10 developmental mechanisms in the following (fixed) order: “the child is biologically ‘preprogrammed’ to have this ability”; “the child has experiences in the womb that give them this ability”; “the child’s body grows and matures (for example, muscles get stronger, child gets taller)”; “the child’s brain changes (for example, brain grows bigger, more or fewer connections between neurons)”; “the child’s senses improve (for example, vision gets sharper, hearing improves)”; “the child observes the objects and the physical world around him or her”; “the child observes the people around him or her”; “the child interacts with the people around him or her”; “people explicitly teach the child how to do this”; “the child actively experiments with how to do this.” This order reflects our rough sense of what might be considered the most innate or biological mechanisms of development, to what might be considered the most learned or social. Participants were also asked to choose among these 10 mechanisms (or to write in another mechanism) to answer the question, “If you had to choose just one, which of the following factors is the most important in the development of capacities for [capacity]?”

Because Study 3 included additional questions probing participants’ theories of developmental mechanisms, to maintain an appropriate level of participant burden we reduced the set of mental capacities from 20 (5 capacities per domain) to 8 (2 capacities per domain). We chose “controlling their emotions” and “reasoning about things” to represent the domain of *cognition and control*; “getting hungry” and “feeling pain” to represent the domain of *bodily sensation*; “learning from other people” and “feeling happy” to represent the domain that we have labeled *social connection*; and “feeling distressed” and “feeling helpless” to represent the domain that we have labeled *negative affect*. Capacities were presented in a random order for each participant.

The 13 target ages for this study were identical to Study 2, with the exception that we added the parenthetical phrase “(at birth)” when referring to newborns.

### Results

#### Perceived Developmental Trajectories.

This approach yielded a very similar picture of US adults’ understanding of the development of human mental life; see Figure 2, Panel B. As in Study 2, participants reported that newborns have very little capacity for *cognition and control* (median score for newborns: 0.00 out of 100 points, *M* = 4.49, *sd* = 12.69), but reported that such capacities increase dramatically over the target age range (*χ*^2^ = 1597.68, *p* < 0.001). Again, participants attributed substantially greater capacities to newborns in the domains that we have labeled *social connection* (median score for newborns: 10.00 out of 100 points, *M* = 30.16, *sd* = 35.74; increase in log-odds: *β* = 2.53, *p* = 0.001) and *negative affect* (median score for newborns: 60.00 out of 100 points, *M* = 55.10, *sd* = 42.02; increase in log-odds: *β* = 2.53, *p* = 0.001); in Study 3, as in Study 2, participants reported substantial increases in the domain that we have labeled *social connection* (*χ*^2^ = 1641.65, *p* < 0.001) and relatively small increases over the target age range in the domain of *negative affect* (*χ*^2^ = 480.78, *p* < 0.001). Finally, as in Study 2, in the domain of *bodily sensation* participants attributed nearly maximal capacity to newborns (median score for newborns: 100.00 out of 100 points, *M* = 89.21, *sd* = 23.20; increase in log-odds: *β* = 4.82, *p* < 0.001) and small increases in capacities for *bodily sensation* over the target age range (*χ*^2^ = 110.98, *p* < 0.001).

Again, visual inspection of Figure 2, Panel B, makes it clear that the shape of these perceived developmental trajectories varied substantially across domains, echoing quite precisely the results of our post-hoc exploratory analyses in Study 2.

#### Intuitive Theories of Development.

Why do US adults believe certain aspects of human mental life, but not others, to be present at birth? What do US adults perceive to be driving the development of capacities for what we have called *bodily sensation, negative affect, social connection*, and *cognition and control*? As a first step in what we hope to be a longer line of work addressing such questions, we asked participants to assess the importance of a variety of possible developmental mechanisms for each of the capacities included in this study. We theorized that when people perceive that a capacity is substantially present at birth, they believe that the capacity is innate, and, conversely, that when people perceive that a capacity develops slowly over years, they believe the capacity is in some sense learned. (For in-depth investigations of laypeople’s intuitions about the innateness of cognitive abilities, motor abilities, and emotions, see Berent et al., 2019, 2020; Wang & Feigenson, 2019.)

We assessed a wide range of potential developmental mechanisms for children’s capacities, and gave participants opportunities to endorse nuanced (perhaps even internally inconsistent) theories of development by having them assess each of these developmental mechanisms for each capacity independently in addition to selecting the “most important” mechanism via forced choice.

**Figure 3.**
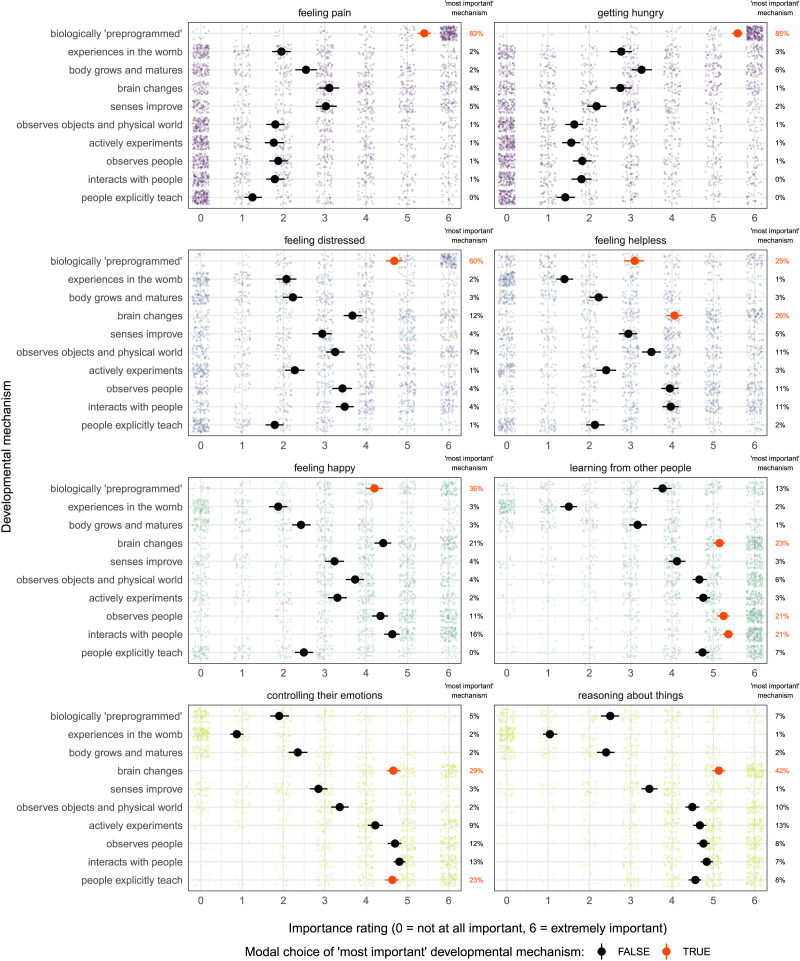
Perceived importance of various mechanisms in the development of four domains of mental life (Study 3); see main text for the full text of each mechanism. Small, lighter points represent individual participants’ ratings of how important each mechanism is in the development of each capacity within a domain (top row: bodily sensation; second row: negative affect; third row: social connection; bottom row: cognition and control). Larger black and red points correspond to mean importance ratings across the sample, and error bars are bootstrapped 95% confidence intervals. On the right side of each panel are the percentages of participants who selected each mechanism as the ‘most important’ driver of development for that capacity, with modal selections (including items within 10% of the mode) in red.

**Figure 4.**
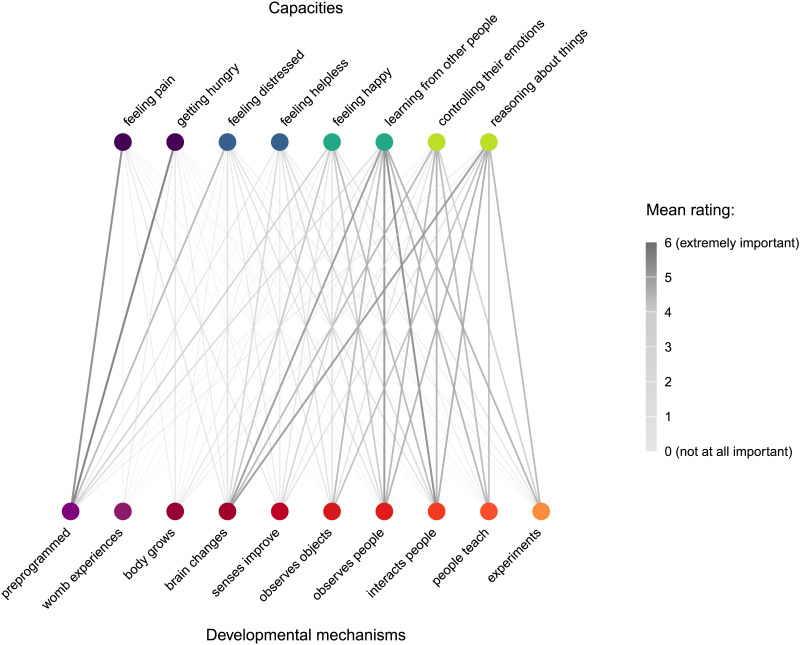
Network graph summarizing the importance of each developmental mechanism (along the bottom) for each capacity (along the top) among US adults (Study 3). Capacities are sorted and color-coded by domain (see Figures 2–3); developmental mechanisms are arranged in the fixed order of presentation (roughly, from what we perceived to be the most innate or biological mechanisms of development, to what we perceived to be the most learned or social). See main text for the full text of each mechanism.

Responses to questions about developmental mechanisms are visualized in Figures 3 and 4.

In terms of selections of the “most important” mechanism driving the development of these capacities between 0–5 years, two explanations—biological preprogramming and brain changes—accounted for most of participants’ selections. Percentage selections and modal responses for all developmental mechanisms are presented in Figure 3; a network graph summarizing the importance of each developmental mechanism for each capacity is presented in Figure 4. (See also Supplemental Materials for a multinomial regression comparing the selection of the most important mechanism across capacities.)

The explanation that “the child is biologically ‘preprogrammed’ to have this ability” was the modal response (or within 10% of the modal response) for 5 of the 8 capacities presented in this study, including all of the capacities chosen to represent the domains of *bodily sensation* (pain, hunger) and *negative affect* (distress, helplessness), as well as one of the two capacities chosen to represent *social connection* (happiness). Consensus on this selection was particularly pronounced for capacities for hunger (85% of participants), pain (83% of participants), and distress (60% of participants).

Meanwhile, the explanation that “the child’s brain changes (for example, brain grows bigger, more or fewer connections between neurons)” was a modal choice for 4 of our 8 capacities, including one of the two capacities chosen to represent *negative affect* (helplessness), one of the two capacities chosen to represent *social connection* (learning from other people), and both of the capacities chosen to represent *cognition and control* (emotional control, reasoning about things). However, consensus about this selection was much lower (23–42% of participants across capacities). Instead, these capacities were characterized by a more diverse selection of mechanisms, including mechanisms that are not as directly tied to biology: For example, when considering what drives the development of a capacity to learn from other people, roughly equal numbers of participants selected brain changes (23%), observations of the social world (21%), and interactions with others (21%); and when considering the capacity for controlling one’s emotions, participants were nearly as likely to select explicit teaching (23%) as they were to select brain changes (29%).

Independent ratings of the importance of each developmental mechanism present a more nuanced—but largely consistent—picture of intuitive theories of development across these domains. See Figures 3–4, and see Supplemental Materials for the full results of the multilevel linear regression analyses briefly presented below.

For capacities in the domain that we have called *bodily sensation* (Figure 3, top row; Figure 4, first and second capacities from the left), “the child is biologically ‘preprogrammed’ to have this ability” stood out as the highest-rated mechanism by far (median score: 6.00 on a scale from 0–6, *M* = 5.50, *sd* = 1.09; comparison to grand mean: *β* = 1.31, 95% CI: [1.26, 1.36], *p* < 0.001), followed by “the child’s brain changes” (median score: 3.00, *M* = 2.93, *sd* = 1.93; comparison to grand mean: *β* = 0.20 [0.15, 0.25], *p* < 0.001), and “the child’s body grows and matures” (median score: 3.00, *M* = 2.90, *sd* = 1.85; comparison to grand mean: *β* = 0.19 [0.14, 0.24], *p* < 0.001). The remaining mechanisms were rated as quite unimportant for capacities in this domain (all medians ≤ 2.50; all *M* ≤ 2.60), with particularly low ratings for “people explicitly teach the child how to do this” (median score: 0.50, *M* = 1.33, *sd* = 1.73; comparison to grand mean: *β* = −0.49 [−0.54, −0.44], *p* < 0.001).

For capacities in the domain of *negative affect* (Figure 3, second row; Figure 4, third and fourth capacities from the left), the two highest-rated mechanisms were again “the child is biologically ‘preprogrammed’ to have this ability” (median score: 4.00 on a scale from 0–6, *M* = 3.89, *sd* = 1.59; comparison to grand mean: *β* = 0.42, 95% CI: [0.36, 0.48], *p* < 0.001) and “the child’s brain changes” (median score: 4.00, *M* = 3.87, *sd* = 1.56; comparison to grand mean: *β* = 0.41 [0.35, 0.47], *p* < 0.001). However, these two biologically inflected explanations were closely followed by explanations that are more social and observational in nature: “the child interacts with the people around him or her” (median score: 4.00, *M* = 3.73, *sd* = 1.64; comparison to grand mean: *β* = 0.35 [0.29, 0.41], *p* < 0.001); “the child observes objects and the physical world” (median score: 4.00, *M* = 3.69, *sd* = 1.63; comparison to grand mean: *β* = 0.19 [0.13, 0.25], *p* < 0.001); “the child observes other people” (median score: 3.50, *M* = 3.38, *sd* = 1.70; comparison to grand mean: *β* = 0.33 [0.27, 0.39], *p* < 0.001). The remaining mechanisms were rated as fairly unimportant for capacities in this domain (median scores: 1.50–3.00; means: 1.73–2.94).

For capacities in the domain that we have called *social connection* (Figure 3, third row; Figure 4, fifth and sixth capacities from the left), the two highest-rated mechanisms were both explicitly social in nature: “the child interacts with the people around him or her” (median score: 5.50 on a scale from 0–6, *M* = 5.00, *sd* = 1.14; comparison to grand mean: *β* = 0.55, 95% CI: [0.50, 0.61], *p* < 0.001) and “the child observes other people” (median score: 5.00, *M* = 4.80, *sd* = 1.19; comparison to grand mean: *β* = 0.46 [0.40, 0.51], *p* < 0.001). These were closely followed by “the child’s brain changes” (median score: 5.00, *M* = 4.78, *sd* = 1.19; comparison to grand mean: *β* = 0.45 [0.39, 0.51], *p* < 0.001). The remaining mechanisms were generally rated as fairly unimportant (median scores: 1.00–4.50; means: 1.69–4.20), with particularly low ratings for “the child has experiences in the womb that give them this ability” (median score: 1.00, *M* = 1.69, *sd* = 1.71; comparison to grand mean: *β* = −1.05 [−1.11, −0.99], *p* < 0.001).

Finally, for capacities in the domain that we have called *cognition and control* (Figure 3, bottom row; Figure 4, last two capacities on the right), many developmental mechanisms were rated as highly important. The highest-rated mechanisms were “the child’s brain changes” (median score: 5.00 on a scale from 0–6, *M* = 4.90, *sd* = 1.19; comparison to grand mean: *β* = 0.62, 95% CI: [0.56, 0.67], *p* < 0.001); “the child interacts with the people around him or her” (median score: 5.00, *M* = 4.82, *sd* = 1.09; comparison to grand mean: *β* = 0.58 [0.53, 0.63], *p* < 0.001); and “the child observes other people” (median score: 5.00, *M* = 4.73, *sd* = 1.10; comparison to grand mean: *β* = 0.54 [0.48, 0.59], *p* < 0.001). Of note, these were closely followed by two mechanisms that were not highly rated in other domains: “people explicitly teach the child how to do this” (median score: 5.00, *M* = 4.60, *sd* = 1.24; comparison to grand mean: *β* = 0.47 [0.42, 0.53], *p* < 0.001); and “the child actively experiments with how to do this” (median score: 4.50, *M* = 4.45, *sd* = 1.27; comparison to grand mean: *β* = 0.40 [0.35, 0.45], *p* < 0.001). The remaining mechanisms were generally rated as fairly unimportant (median scores: 0.00–4.00; means: 0.96–3.93), with particularly low ratings for “the child has experiences in the womb that give them this ability” (median score: 0.00, *M* = 0.96, *sd* = 1.35; comparison to grand mean: *β* = −1.27 [−1.32, −1.22], *p* < 0.001).

In sum, we would characterize participants’ intuitive theories as follows. For capacities in the domain that we have called *bodily sensation*, there was broad consensus across participants that development between ages 0–5 years is driven almost exclusively by biological forces, particularly some form of biological “preprogramming.” For all other capacities, participants viewed development as the result of a combination of biological forces (including “preprogramming”) and experience in the physical and social world. In the domain that we have called *negative affect*, responses suggested that passive observation of the world might be considered sufficient to drive early development. In contrast, in the domains that we have called *social connection* and *cognition and control* there were some suggestions that a child must interact with the world in order to develop (for example, via social exchanges with other people or by conducting their own “experiments”).

## DISCUSSION

In a series of three large-scale studies, we identified four core capacities that anchor US adults’ conceptual representations of the development of human mental life from birth through early childhood (Study 1); measured US adults’ perceptions of these capacities at birth and charted how they are perceived to change over the first five years of a child’s life (Studies 2–3); and explored the intuitive theories that underlie the observed differences in the perceived developmental trajectories of these four domains (Study 3).

We found that participants considered a core capacity for *bodily sensation* (e.g., hunger, pain) to be present from very early in an infant’s life—in many cases, “preprogrammed” and present at birth—leaving relatively little room for development over childhood. In contrast—and in line with previous work on adults’ intuitions about cognitive development (Berent et al., 2019; Wang & Feigenson, 2019)—participants considered capacities for *cognition and control* (e.g., planning, self-control) to be largely absent at birth and to develop steadily and gradually over the first five years of life, driven primarily by driven primarily by observation of and interactions with the physical and social world.

Beliefs about the development of the more social-emotional aspects of mental life—what we have called *negative affect* (e.g., distress, frustration) and *social connection* (e.g., happiness, love, learning from others)—were intermediate between the extremes of *bodily sensation* and *cognition and control*. In the aggregate, participants considered capacities for *negative affect* and *social connection* to be present to some degree from birth (consistent with recent findings on the perceived “innateness” of emotions, Berent et al., 2020) but to continue developing rapidly over the first two years of life. Likewise, participants reported that development in these capacities was driven by a combination of biological forces (including “preprogramming”) and experience in the physical and social world. Both in their perceptions and in their explanations of development, participants’ understanding of *negative affect* were somewhat more similar to their responses to capacities for *bodily sensation*, while their responses to capacities for *social connection* more closely resembled their understanding of *cognition and control*. We note that attributions of what we have labeled *negative affect* and *social connection* were particularly variable across individual participants, especially when it came to perceptions of the capacities of newborns; see Figure 2. This suggests that beliefs about development in these domains are especially sensitive to personal experience, social-cultural context, explicit education, or other forms of input.

### Comparison to Previous Work

In some respects, the results of these studies resonate well with previous work on mind perception among US adults. The factors that we refer to as *bodily sensation* and *cognition and control* highlight a sharp distinction between embodied, physiological experiences and the more cognitive and agentic aspects of mental life experiences—reminiscent of previous distinctions between “experience” and “agency” (Gray et al., 2007), between “body” and “mind” (Weisman et al., 2017b, 2021), and between “affect” and “regulation” (Malle, 2019). These two factors were clearly evident in all of the factor solutions we examined, providing further evidence that this distinction is a particularly important aspect of how US adults reason about mental life. (See also Weisman et al., 2021, for evidence that a distinction between bodily sensation and cognition is held in common across diverse cultural settings.)

However, these studies also highlight one way in which reasoning about the development of human mental life may diverge from reasoning about mental life in general: In their assessments of the mental lives of infants and children at different ages, US adults differentiated between what we have called *social connection* (e.g., capacities for excitement, humor, and love) and what we have called *negative affect* (e.g., capacities for distress, helplessness, and frustration). These two factors have no obvious precedents in previous studies of mind perception. In the agency-experience framework (Gray et al., 2007), *negative affect* would likely fall under the umbrella of “experience,” but *social connection* seems to combine aspects of both “experience” (e.g., happiness, sadness) and “agency” (e.g., learning, recognition); similarly, in Malle’s (2019) framework, *negative affect* clearly resonates with “affect,” but *social connection* seems to combine aspects of both “regulation” and “reality interaction.” Meanwhile, in the body-heart-mind framework (Weisman et al., 2017b), *social connection* and *negative affect* might both be considered part of the social-emotional domain of the “heart.”

This difference from previous work could have many (or multiple) causes, including the wider range of capacities included in this study (in particular, the inclusion of capacities for feelings of helplessness, frustration, annoyance, neglect, loneliness, boredom, confusion, and being overwhelmed; see Supplemental Materials); the emphasis on how mental capacities develop over time; or, more broadly, the exclusive focus on humans as the targets of mind perception. Indeed, one exciting line of work focusing on how laypeople think about other humans’ mental states has surfaced a robust conceptual architecture in which mental states are organized in three dimensions: “rationality”, capturing a distinction between cognitive and affective states; “social impact”, capturing the degree to which mental states involve social relationships; and “valence”, capturing the distinction between positive and negative states (Tamir et al., 2016; Tamir & Thornton, 2018; Thornton & Tamir, 2020). The domains that we have called *social connection* and *negative affect* align rather well with these latter two dimensions, capturing a conceptual distinction between the degree to which certain capacities shape and are shaped by interpersonal interactions and social relationships vs. the degree to which certain capacities shape and are shaped by firsthand experiences of negative valence. Based on the findings of Studies 2 and 3, we further propose that *social connection* and *negative affect* emerged as distinct factors not only because they demarcate different aspects of mental life, but also because participants believed these two aspects of mental life develop differently: i.e., that they are present to different degrees at birth, develop at different rates, and are driven by different developmental mechanisms.

What do these studies reveal about participants’ understanding of the contributions of nature vs. nurture to the development of human mental life? One consistent finding across these studies is that participants attributed many capacities to newborn infants, at least to a moderate degree. In addition to *bodily sensation* and *negative affect*—obvious aspects of a newborn’s experience of the world—participants also reported that newborns had fairly substantial social-cognitive abilities, including, critically, “learning from other people”. Indeed, in Study 3 participants considered biological “preprogramming” to play a rather important role in the ability to learn from other people, and to have some non-trivial impact even on the most purely “cognitive” ability included in that study, reasoning about things (see Figure 3). Rather than “intuitive empiricists” (Berent et al., 2021; Wang & Feigenson, 2019) or “intuitive nativists”, then, US adults might be better described as “intuitive constructivists” who viewed newborns as predisposed and innately equipped to learn from the people around them. Further work on this topic would do well to integrate the cognitive processing mechanisms featured in the current studies with epistemic states that have been the focus of past research on laypeople’s beliefs about the innateness of “cognitive” abilities (Berent et al., 2019; Wang & Feigenson, 2019) to paint a more comprehensive picture of the ways in which intuitive theories of development do and do not align with scientific theories such as nativism, empiricism, and constructivism.

### Limitations

One important limitation of the current studies is that they relied exclusively on samples of US adults recruited via the crowdsourcing platform Amazon Mechanical Turk (MTurk). In other lines of work on mind perception using similar bottom-up approaches to identifying latent conceptual structures, MTurk samples have yielded factor solutions very similar to more representative samples of general population US adults collected in person in public settings (e.g., the Department of Motor Vehicles, Weisman et al., 2021); given this, we would predict that the current findings would be replicated in more representative samples of predominantly White, middle-class US adults. Of great interest and importance is the question of how beliefs and intuitive theories about the development of mental life do and do not vary across cultural, ethnic, racial, socioeconomic, educational, generational, and other dimensions, both within the US and across national boundaries. Echoing recent work comparing concepts of mental life across diverse cultural settings (Weisman et al., 2021), we speculate that representations of the development of the more affective and emotional aspects of mental life is where the largest differences across cultural and demographic groups would be found, while other aspects of these beliefs might be held in common—but this is of course a question deserving of careful empirical investigation.

Related to the issue of the cultural specificity of our findings is the issue of the specificity of our stimuli. In selecting photos for each target age, we aimed to have photos of the same (or very similar-looking) children at all target ages so as to emphasize the question of the development of mental capacities over the course of an individual child’s early life. We chose to illustrate each target age with two photos in order to emphasize that we were interested in children’s mental capacities in general, rather than the experiences of a particular child in a particular moment. We included one boy and one girl at each target age in order to generalize across potential perceived sex/gender differences in children’s early mental life. We used photographs of two (apparently) White, non-Hispanic children, for three reasons: (1) in order to avoid introducing spurious differences across target ages by confounding a target’s age and race/ethnicity; (2) in order to match the anticipated race/ethnicity of the majority of participants on MTurk; and (3) because of the limited set of high-resolution photographs of individual children of color at different points of development available to us. Conducting similar studies of adults’ perceptions of the development of mental life with more ethnically and racially diverse participants, and examining the effects of sex, gender, race, and ethnicity on adults’ perceptions of children’s mental life, will be important next steps in this line of research.

On a similar note, although our instructions emphasized to participants that we were interested in their assessment of children in general, rather than the depicted children in particular, it is nearly inevitable that the features of the particular photographs used as stimuli—not just race and ethnicity, but also facial expressions, attractiveness, cues to social class, and so on—would guide participants’ responses. Future research might employ a variety of approaches to address this concern, such as constructing a much larger and more diverse pool of visual stimuli and varying the subset of stimuli provided across participants; blurring children’s facial expressions; blurring or removing the background of the photographs; or removing all visual stimuli from the study (likely more appropriate for parents of young children than for a general population study).

One of the most interesting future directions for this line of research will be to assess how the experience of raising a child might change caregivers’ representations of and intuitive theories about the development of mental life across infancy and early childhood. Unfortunately, the current datasets are well-suited to these explorations. Our samples tended to include more non-parents than parents, and to include a wide range of parents (including, e.g., parents of children ranging in age from newborns to middle-aged adults). We also strongly suspect that parent status in MTurk samples is likely confounded with many demographic variables (e.g., age, wealth, education level, geographical location within the US), most of which we did not measure in the current samples. For these reasons, we have opted not to include any analyses of parents vs. non-parents in the current manuscript, because we do not want to mislead readers with potential spurious results from these datasets. However, we consider careful comparisons of parents and non-parents, as well as close longitudinal explorations of the effects of caregiving on beliefs about the development of mental life, to be topics warranting further research.

Finally, while our current analytical approach identifies key patterns in these developmental trajectories, future work could benefit from employing functional data analysis methods, such as functional principal components analysis (fPCA), which might capture smooth, continuous variations in temporal patterns and better account for the inherent time-dependency in developmental data (Ramsay & Silverman, 2002). Such an approach might identify clusters of mental capacities that hang together not only in terms of overall covariance, but also in terms of the perceived timing and rate of development, or in terms of other temporal dynamics that our current approach might not detect. Employing such techniques would be an exciting next step in this line of research.

### Implications for Caregiver–Child Interactions

How might the distinctions between capacities for *bodily sensation, negative affect, social connection*, and *cognition and control* inform caregiver–child interactions? Drawing on theoretical proposals from other work in mind perception (Weisman et al., 2017b), we would argue that what gives meaning to the four factors documented in the current studies is the possibility that they correspond to substantially different causal explanatory frameworks for interpreting, predicting, and responding to the behaviors of a toddler—i.e., distinct lenses through which a caregiver might view and understand a child in a given moment. Consider a caregiver attempting to navigate the difficult situation of a toddler throwing a “temper tantrum.” Through the lens of *bodily sensation*, the caregiver might understand the child as driven by basic biological needs, which could draw the caregiver’s attention to the possibility that the child is hungry, fatigued, injured, or ill, and focus the caregiver’s behaviors on the child’s physical wellbeing. Through the lens of *negative affect*, the caregiver might instead understand the child as a moral patient who deserves to be spared unpleasant and harmful experiences, drawing attention to the possibility that the child is upset or afraid and motivating the caregiver to provide comfort. Through the lens of *social connection*, the caregiver might understand the child as a social partner, interpreting the child’s distress as an indication that the relationship needs repair and seeking to remedy the disruption. And through the lens of *cognition and control*, the caregiver might understand the child as an intentional agent who has been thwarted in the pursuit of certain goals; the caregiver might offer the child assistance toward the goal, or age-appropriate techniques to regulate the child’s negative emotions. Of course, by the time a human reaches adulthood, all of these explanatory frameworks are reasonable modes of interpreting an individual’s behavior, because human adults are biological animals, moral patients, social partners, and intentional agents. But when faced with a distressed toddler—especially when limited verbal skills require caregivers to make guesses what kind of care is required—these four modes of understanding might have important consequences for caregivers’ responses and, in turn, for children’s outcomes.

In other words, adults’ intuitive theories are likely to have important consequences for the children in their care (see also Haimovitz & Dweck, 2016; Hembacher & Frank, 2020; Mukhopadhyay & Yeung, 2010). We suspect that intuitive theories of the development of mental life could play a particularly important role in the quality of caregiver–child relationships in the first few years of a child’s life. During this period, caregivers often must *infer* what children think and feel. These inferences critically depend on the adult’s understanding of what the child is and is not capable of, and what scaffolding it would take for that child to become more capable. There is ample evidence that representations of children’s mental lives—including their sensations, perceptions, emotions, cognitive abilities, executive function, and social skills—play a critical role in guiding adults’ expectations of a child, their reactions to that child’s actions, and the nature and development of their relationship with the child (Feldman & Reznick, 1996; Vreeswijk et al., 2012). At a high level, caregivers’ “mind-mindedness”—that is, their capacity to form an accurate representation of their child’s internal states and to use this representation to guide their caregiving behavior—is positively associated with children’s wellbeing, including attachment security, theory of mind, and ultimately, school readiness (Bernier et al., 2017; Meins et al., 2012, 2013). The current studies reframe these and similar findings on mind-mindedness as reflecting not only an attentional focus on the part of the caregiver but also the intuitive theories that shape caregivers’ interpretations of and responses to children’s behaviors. Our results further highlight the possibility that differentiating between different aspects of “mind” and developmental shifts in mind perception could push this highly influential research program in important new directions. For example, perceptions of a child’s capacities in different aspects of mental life might influence caregivers’ tendencies to demonstrate mind-mindedness in some contexts more than others (e.g., when the child is experiencing physical discomfort vs. emotional distress, when the child is working on a task vs. attempting to strengthen a social bond), or at one developmental stage more than another (e.g., mind-mindedness may be more apparent with respect to *bodily sensation* in infancy vs. *cognitive control* in later childhood).

We conclude by highlighting three related, but meaningfully distinct, approaches to studying the connections between adults’ intuitive theories, parenting behaviors, and children’s wellbeing. We introduce these approaches as open questions ripe for future research—and critical next steps for translating this basic research into useful interventions to promote positive caregiver–child relationships and child wellbeing.

First, are the beliefs and intuitive theories documented here accurate with respect to the current scientific understanding of human development? Comparing participants’ intuitions to empirical findings from scientific studies of child development has illuminated the ways in which lay people’s reasoning about cognitive development (Berent et al., 2019; Wang & Feigenson, 2019) and about emotions (Berent et al., 2020) has proved a useful way of illuminating biases and misconceptions in laypeople’s beliefs about innateness. The primary goal of the current studies was to characterize how US adults think about development, rather than to generate precise estimates of the age at which lay people consider each of these capacities to “come online”; nonetheless, each of these studies provides estimates of participants’ beliefs about children’s capacities at different ages, and the collection of these studies provides some indication of how stable these estimates are across different methods of administration; see Supplemental Materials for direct comparisons of developmental trajectories across studies at the level of the individual capacity (Figure S9) and the domain (Figure S10). Finding creative ways to determine the ground truth of the development of the range of capacities included in the current studies—many of which are phenomenal experiences—will be a particular challenge to this endeavor, but would be both deeply fascinating and of practical interest to predicting downstream consequences for children. All else being equal, we would predict that caregivers with more accurate representations of children’s developing minds would make more accurate inferences about their own children and interact with their children in ways that are more developmentally appropriate and better-suited to scaffolding the child’s development (see, e.g., List et al., 2021).

A second question that could drive extensions of the current work is: How do these beliefs and intuitive theories impact caregivers’ affective experience of caregiving? On the whole, we would predict that educating parents about infants’ mental capacities and their rapid development might spark increased curiosity about child development, with positive downstream consequences for parental well-being as well as the quality of parent-child interactions. We note, however, that even beliefs that do not align with modern scientific understandings of development—e.g., the belief that newborns love and care for their siblings, the belief that toddlers might remember previous lives—may play motivational roles in sustaining caregivers’ interest in their children’s minds and encouraging positive parenting behaviors; and, conversely, that some accurate beliefs—e.g., the belief that children will eventually forget most of what happens in infancy and toddlerhood—could be demotivating for caregivers.

This brings us to the third orienting question for extensions of this work: What set of folk beliefs and intuitive theories are most adaptive, i.e., most effective at facilitating developmentally appropriate caregiving behaviors and positive outcomes for children? This question is of course deeply related to the issues of accuracy and affective experience just described; for example, under-attributions of any aspect of mental life on the part caregivers might result in reduced opportunities for interaction and psychosocial stimulation for the children in their care. Aside from sparking caregivers’ interest in their children, certain over-attributions of mental life might foster among caregivers the very behaviors that create and nurture these nascent capacities. For example, crediting an infant with more of a capacity for *social connection* than may be warranted by empirical research could lead a caregiver to engage in the warm, reciprocal interactions that jumpstart the development of early social skills. Falsely assuming that a toddler is capable of feeling and understanding complex emotions like guilt and embarrassment might lead the caregiver to use the kind of language that scaffolds the development of such sophisticated social-emotional representations. Conversely, over-attributions of the capacity for *cognition and control* might result in caregivers attributing overt intentions to annoy or upset their caregiver and could lead caregivers to be overly punitive in their response to what they perceive as deliberate “acting out” (see Bugental et al., 2002). We offer these speculative examples to illustrate the many possibilities that adopting the lens of adaptivity could open for researchers and interventionists interested in the effects of caregivers’ intuitive theories on their children’s well-being—but, of course, such interventions would require extensive research focused on caregivers rather than general-population adults, carefully situated within a particular setting so as to promote caregiving behaviors that are appropriate for the children in question.

Whether focused on improving accuracy, affective experience, or adaptiveness, critical next steps in this line of research will involve assessing which aspects of adults’ beliefs and theories about the development of mental life facilitate the kinds of caregiving behaviors that are most beneficial for children according to experts, most appropriate in a given cultural setting, or most in line with caregivers’ own values and goals. Identifying the antecedents of individual and group-wise differences in these beliefs—especially those that might lead caregivers to over- or under-estimate a child’s abilities and thus misinterpret the child’s behaviors, miss opportunities to support the child’s development, or become overly concerned about their child’s developmental progress—will be an especially important step for such clinically-focused applications, including interventions to reduce risk for child abuse and neglect (King et al., 2021). As is the case in many domains (Weisman & Markman, 2017), interventions that leverage the power of intuitive theories to encourage positive and responsive caregiving behaviors hold great promise for creating meaningful and lasting behavioral changes in caregiver behavior, with important consequences for child wellbeing.

## Funding Information

Funding was provided by the William R. and Sara Hart Kimball Stanford Graduate Fellowship (Weisman); the National Science Foundation (Weisman and King: Graduate Research Fellowships; Humphreys: CAREER 2042285), the National Institutes of Health (King: F32 HD105385), and the Jacobs Foundation (Humphreys: Early Career Research Fellowship 2017-1261-05).

## Author Contributions

Kara Weisman: Conceptualization; Data curation; Formal analysis; Investigation; Methodology; Software; Validation; Visualization; Writing – original draft; Writing – review & editing. Lucy S. King: Conceptualization; Data curation; Methodology; Writing – review & editing. Kathryn Humphreys: Conceptualization; Funding acquisition; Methodology; Project administration; Supervision; Writing – original draft; Writing – review & editing.

## Data Availability statement

Study materials, preregistrations, data, analysis scripts, and links to the fully reproducible manuscript and supplemental materials are openly available via the project’s Open Science Framework page (https://osf.io/xrznd/; DOI: 10.17605/OSF.IO/XRZND).

## Supplementary Material


