# Supplementary material for: Beliefs About the Development of Mental Life
Source: Open Mind (Camb). 2025 Apr 22;9:515–39. doi: 10.1162/opmi_a_00200 (PMC12058329; doi:10.1162/opmi_a_00200)
Supplement: Supplementary file 1 [file opmi-09-515-s001.pdf]

Beliefs about the development of mental life: Supplemental Materials

Kara Weisman
Department of Psychology, University of California, Riverside

Lucy S. King
Department of Psychology and Human Development, Vanderbilt University

Kathryn Humphreys
Department of Psychology and Human Development, Vanderbilt University

Author Note

Kara Weisman: https://orcid.org/0000-0002-3706-7554. Lucy S. King: https://orcid.org/0000-0002-2552-0614. Kathryn Humphreys: https://orcid.org/0000-0002-5715-6597.

Weisman, King, and Humphreys were affiliated with the Department of Psychology at Stanford University at the time of data collection. Weisman was affiliated with the Department of Psychology at the University of California, Riverside, at the time of submission, and is now affiliated with the Department of Psychology at Harvard University. King and Humphreys are now affiliated with the Department of Psychology and Human Development at Vanderbilt University.

Study materials, preregistrations, data, analysis scripts, and links to the fully reproducible manuscript and supplemental materials are openly available via the project's Open Science Framework page (https://osf.io/xrznd/; DOI: 10.17605/OSF.IO/XRZND). Funding was provided by the William R. and Sara Hart Kimball Stanford Graduate Fellowship (Weisman); the National Science Foundation (Weisman and King: Graduate Research Fellowships; Humphreys: CAREER 2042285), the National Institutes of Health (King: F32 HD105385), and the Jacobs Foundation (Humphreys: Early Career Research Fellowship 2017-1261-05).

Correspondence should be addressed to Kara Weisman at kgweisman@gmail.com.

Contents

Extended methods **2**
Study 1 2
Study 2 5
Study 3 7

Extended results **9**
Study 1 9
Study 2 21
Study 3 25

Study comparison **33**

Selection of capacities: Comparison to previous studies **33**

References **41**

In a series of large-scale studies we assessed how US adults conceptualize the development of the human mind over the first five years of life. We identified four core capacities that are perceived by US adults to develop over human infancy and early childhood, *bodily sensation*, *negative affect*, *social connection*, and *cognition and control* (Study 1); charted how these different aspects of mental life are perceived to change over the first five years of a child's life (Studies 2-3); and explored the intuitive theories that underlie the observed differences in the perceived developmental trajectories of these four aspects of mental life (Study 3).

In these Supplemental Materials, we provide extended information about the methods and results presented in the main paper, present additional results, and provide an item-by-item comparison of the capacity items used here to items used in previous work on mind perception (Gray et al., 2007; Malle, 2019; Weisman et al., 2017b, 2017a).

Note: This is a fully reproducible manuscript, written in RMarkdown using the "knitr" and "bookdown" packages for R (Xie, 2016, 2021, 2024). The full, editable script for all analyses, figures, and tables (here and in the main text) is available in the .Rmd file that generated this document (supplement-main.Rmd), which can be found in the following GitHub repository: https://github.com/kgweisman/baby_mental_life_ms.

Extended methods

Methods, inclusion/exclusion criteria, and analyses marked as "preregistered" were preregistered on the Open Science Framework (OSF) website (Study 1: https://osf.io/e6ajh; Study 2: https://osf.io/j72dg; Study 3: https://osf.io/xh8ce)).

All materials used in these studies are available in the following OSF repository: https://osf.io/xrznd.

All studies were approved by the Stanford University Research Compliance Office (Human Subjects IRB).

Study 1

Participants

301 US adults participated via Amazon Mechanical Turk (MTurk) in July–August 2018. To be eligible for the study, participants were required to have gained approval for at least 95% of their previous work on MTurk, with at least 50 HITs completed; have MTurk accounts based in the US; indicate that they were between the ages of 18-45 years; and report that their English abilities were either "advanced" or "superior" (not "novice," "intermediate," or decline to provide information about English ability). Participants were paid $2.00 for approximately 10-15 minutes of their time.

According to self-report, participants ranged in age from 19-45 years (M = 31.37 years, sd = 5.75 years) and included more men (59%) than women (41%; <1% of participants identified as some other gender or declined to disclose). Participants predominantly identified as White (66%; <15% identified as any other race/ethnicity, identified as more than one race/ethnicity, or declined to disclose). 51% of participants had obtained at least a Bachelor's degree. 44% of participants indicated that they were parents.

An additional 77 participants completed the study but were excluded for failing one or more (out of three) embedded attention checks (e.g., failing to choose "34" in response to the question "Please select 34") or for indicating via a multiple choice question at the end of the survey that they had not paid attention, had not avoided distractions, or had not taken the study tasks seriously.

Materials and procedure

After completing a screening questionnaire (in which participants provided information about their gender/sex, age, and English abilities) and providing informed consent, participants were provided with an overview of the study, as follows: "In this study, we will ask you about the mental capacities of children of different ages, from newborns to 5-year-olds. We will show you example photos of children at three of the following ages to remind you of what children look like at that age, but please think about children of that age in general when answering these questions. There are 60 brief questions for each of the three ages (for example, 'To what extent is a newborn capable of feeling happy?'), followed by a few demographic questions about yourself. We are interested in your opinions about these questions. Please do not look up extra information about these questions—we just want to know what you think." Participants were shown an array of pictures of children at several target ages between 0-5 years; see "Target ages and accompanying photographs," below.

Participants then proceeded through three trials in which they assessed the mental lives of children at three target ages: birth, 9 months, and 5 years. On each trial, they were shown two representative photographs of children at the target age (with the photograph labeled "newborns," "9-month-olds," or "5-year-olds"), and asked to answer the following question for 60 capacities: "To what extent is a [newborn/9-month-old/5-year-old] capable of [this capacity]?" Participants responded on a sliding scale from 0 (labeled as "not at all capable") to 100 (labeled as "completely capable").

On the final page of the survey, participants were asked to provide additional demographic information.

Capacities The 60 capacities included in Study 1 were drawn from several areas of previous research. Our goals in selecting items were to: (1) balance the representation of the conceptual organization underlying mental capacity attributions in general, as identified in previous work (Gray et al., 2007; Malle, 2019; Weisman et al., 2017b, 2017a, 2018); (2) add items relevant to early development, as identified by existing measures of temperament and behavior in early life (Putnam et al., 2006; Rothbart, 1978); and (3) assess the broadest range of mental capacities within financial and participant burden constraints, as identified by extensive discussions among the authors drawing on our own research and clinical experience with infants and young children.

This process yielded a list of 60 capacities. Roughly half of this list (29 of 60 items) were drawn from (or closely based on) the items included in Weisman et al.'s recent studies (Weisman et al., 2017b): "being aware of things", "feeling calm", "feeling embarrassed", "feeling guilty", "feeling happy", "feeling love", "feeling pain", "feeling pleasure", "feeling pride", "feeling sad", "feeling safe", "feeling scared", "feeling tired", "getting angry", "getting hungry", "getting hurt feelings", "having goals", "having self-control", "having thoughts", "having wants and desires", "hearing sounds", "making choices", "planning", "reasoning about things", "recognizing others' emotions", "recognizing somebody else", "remembering things", "seeing", and "telling right from wrong". By extension, the list included close variants on 14 of the 20 capacities evaluated in Gray et al.'s original study (Gray et al., 2007).

The other half (31 of 60 items) were novel items, not used in previous studies of mind perception, but common in clinical assessments of infant temperament and behavior: "being afraid of somebody", "being angry at somebody", "being comforted by physical touch", "calming themselves down", "controlling their emotions", "detecting danger", "feeling annoyed", "feeling bored", "feeling confused", "feeling distressed", "feeling excited", "feeling frustrated", "feeling helpless", "feeling hopeless", "feeling lonely", "feeling neglected", "feeling overwhelmed", "feeling physically uncomfortable", "feeling textures (for example, 'smooth', 'rough')", "feeling thirsty", "feeling too hot or too cold", "feeling worried", "finding something funny", "focusing on a goal", "getting pleasure from music", "imagining things", "learning from other people", "listening to somebody", "loving somebody", "thinking before they act", and "understanding what somebody else is thinking".

See "Selection of capacities: Comparison to previous studies" for an item-by-item comparison of the capacities used here to those used in previous studies of mind perception (Gray et al., 2007; Malle, 2019;

Weisman et al., 2017b, 2017a).

Capacities were presented in a random order each time they were displayed (i.e., order varied across both target ages and participants).

Target ages and accompanying photographs In planning this series of studies, we selected 13 target ages based on the developmental milestone ages identified by the CDC (Centers for Disease Control and Prevention, 2021) with the addition of the American Academy of Pediatrics' recommendations for pediatrician visits before 2 months (American Academy of Pediatrics, 2018): birth, 4 days, 1 month, 2 months, 4 months, 6 months, 9 months, 12 months, 18 months, 2 years, 3 years, 4 years, and 5 years. We limited Study 1 to just the youngest, median, and oldest target ages—presented to participants as "newborns," "9-month-olds" and "5-year-olds"—in order to maximize the number of capacities included in the study without over-burdening participants.

In selecting photos for each target age, we aimed to have photos of the same (or very similar-looking) children at all target ages so as to emphasize the question of the development of mental capacities over the course of an individual child's early life. We chose to illustrate each target age with two photos in order to emphasize that we were interested in children's mental capacities in general, rather than the experiences of a particular child in a particular moment. We also presented participants with instructions that underscored this point (see above). We included one boy and one girl at each target age in order to generalize across perceived sex/gender differences in children's early mental life. We used photographs of two (apparently) White, non-Hispanic children, for three reasons: (1) In order to avoid introducing spurious differences across target ages by confounding a target's age and race/ethnicity; (2) In order to match the anticipated race/ethnicity of the majority of participants on MTurk; and (3) Because of the limited set of high-resolution photographs of individual children of color at different points of development currently available to us. Conducting similar studies of adults' perceptions of the development of mental life with more ethnically and racially diverse participants, and examining the effects of sex, gender, race, and ethnicity on adults' perceptions of children's mental life, will be critical next steps in this line of research.

Target ages were presented in chronological order, with participants first assessing all 60 capacities for newborns, then 9-month-old infants, and finally 5-year-old children.

Analysis plan

Traditional exploratory factor analysis techniques are not equipped to account for within-participant variance alongside between-participant variance. To conduct the EFAs reported in Results, we proceeded as if each participant's ratings of the 60 mental capacities for each of the three target ages were a distinct source of data; in other words, as if we had 903 participants assessing one target age each, instead of 301 participants assessing three target ages each. See (Weisman et al., 2017b, 2021), for similar treatments of within-subjects data in the context of EFA; but see also (Owen & Wang, 2016) for a bi-cross-validation technique, which might be adapted to address this repeated-measures design by structuring cross-validation folds to exclude all observations from a given participant within a given fold. (We thank a helpful reviewer for this suggestion.)

We used Pearson correlations to obtain minimal residual solutions and applied an oblique transformation (oblimin) to the resulting factor structures, using the "psych" package for R (Revelle, 2021).

In order to determine how many factors to retain, we examined the results of three factor retention protocols: (1) Parallel analysis, which compares the observed correlation structure to the correlation structure arising from random datasets of the same size; (2) Minimizing the Bayesian Information Criterion (BIC), which is one method of optimizing both goodness of fit and parsimony; and (3) A set of factor retention criteria that have been used in Weisman et al.'s previous work (Weisman et al., 2017b), in which they retained factors with eigenvalues greater than 1.0, which individually accounted for greater than 5% of the shared variance before transformation or rotation, and which were the "dominant" factor (the factor with the strongest absolute factor loading) for at least one mental capacity after transformation (a factor had to meet all three of these criteria in order to be retained). Our interpretation of how best to characterize

our dataset (i.e., how many factors we observe) was guided by the degree of consensus among these three protocols and the interpretability of the retained factors under each protocol.

Parallel analysis suggested a 4-factor solution (accounting for 72% of the total variance); minimizing BIC suggested a 6-factor solution (accounting for 74% of the total variance); and Weisman et al.'s (2017) factor retention criteria suggested a 2-factor solution (accounting for 68% of the total variance). We present the 4-factor solution in the main text because it was of intermediate size and because we judged the factors it revealed to be intelligible and of theoretical interest; we present all three solutions in these Supplemental Materials.

Study 2

Participants

304 US adults participated via MTurk in August 2018. Eligibility requirements were identical to Study 1. Participants were paid $2.90 for approximately 15-20 minutes of their time. In response to the overrepresentation of men in Study 1, in Study 2 we recruited men and women through separate "Human Intelligence Tasks" (HITs).

According to self-report, participants ranged in age from 19-45 years (M = 32.14 years, sd = 6.32 years) and (per our recruitment strategy) included roughly equal numbers of men (51%) and women (49%). Participants predominantly identified as White (75%; <11% identified as any other race/ethnicity, identified as more than one race/ethnicity, or declined to disclose). 50% of participants had obtained at least a Bachelor's degree. 41% of participants indicated that they were parents.

An additional 90 participants completed the study but were excluded for failing one or more (out of four) attention checks or indicating that they had not paid attention (see Study 1).

Materials and procedure

After completing a screening questionnaire (in which participants provided information about their gender/sex, age, and English abilities) and providing informed consent, participants were provided with an overview of the study that was nearly identical to that used in Study 1.

Participants then proceeded through 13 trials in which they assessed the mental lives of children at 13 target ages: birth, 4 days, 1 month, 2 months, 4 months, 6 months, 9 months, 12 months, 18 months, 2 years, 3 years, 4 years, and 5 years. On each trial, they were shown two representative photographs of children at the target age, and asked to answer the following question for 20 capacities: "To what extent is a [newborn/4-day-old/etc.] capable of [this capacity]?" As in Study 1, participants responded on a sliding scale from 0 (labeled as "not at all capable") to 100 (labeled as "completely capable").

On the final page of the survey, participants were asked to provide additional demographic information.

Capacities In order to ask participants to assess a more fine-grained array of target ages without undue participant burden, we limited our list of capacities to 20 of the 60 capacities used in Study 1. To identify this list, we drew on the results of Study 1, selecting 5 items for each of the 4 factors identified there. For each factor, we aimed to select items that loaded strongly on that factor, did not cross-load strongly on other factors, were sufficiently distinguishable from each other in meaning (i.e., not overly redundant), and captured our qualitative understanding of the latent construct that each factor corresponded to.

To represent *bodily sensation*, we chose the following items: "getting hungry", "feeling pain", "feeling tired", "feeling physically uncomfortable", and "hearing sounds". In the oblimin-transformed 4-factor solution from Study 1 all of these items had loadings ≥ 0.75 on this factor, and absolute loadings ≤ 0.21 on the other three factors.

To represent *negative affect*, we chose the following items: "feeling distressed", "feeling lonely", "feeling frustrated", "feeling helpless", and "feeling overwhelmed". In Study 1 all of these items had loadings ≥ 0.35 on this factor, and absolute loadings ≤ 0.49 on the other three factors. As these numbers suggest, this was a difficult factor to represent, because there were no items that loaded very strongly on the factor (highest loading: 0.45) and because the highest-loading items tended to cross-load on other factors as well. Nonetheless, we decided to try to represent this latent construct because of its theoretical interest and potential clinical importance.

To represent *social connection*, we chose the following items: "feeling excited", "finding something funny", "loving somebody", "learning from other people", and "feeling happy". In the oblimin-transformed 4-factor solution from Study 1 all of these items had loadings ≥ 0.79 on this factor, and absolute loadings $\leq$ 0.16 on the other three factors.

Finally, to represent *cognition and control*, we chose the following items: "planning", "having self-control", "reasoning about things", "controlling their emotions", and "telling right from wrong". In the oblimin-transformed 4-factor solution from Study 1 all of these items had loadings ≥ 0.91 on this factor, and absolute loadings ≤ 0.11 on the other three factors.

Capacities were presented in a random order each time they were displayed (i.e., order varied across both target ages and participants).

Target ages and accompanying photographs The target ages for this study (birth, 4 days, 1 month, 2 months, 4 months, 6 months, 9 months, 12 months, 18 months, 2 years, 3 years, 4 years, and 5 years) were based on the developmental milestone ages described in Study 1. The pairs of photos used to illustrate each target age were identical to those used in Study 1; see Study 1, Methods, for more information.

As in Study 1, target ages were presented in chronological order, with participants first assessing all 20 capacities for newborns, then 4-day-old infants, then 1-month-old infants, and so forth.

Analysis plan

Preregistered confirmatory analysis of conceptual structure Based on the results of Study 1, we predicted that the correlational structure of participants' responses would reveal four underlying constructs corresponding to the four factors we identified in Study 1: *bodily sensation*, *negative affect*, *social connection*, and *cognition and control*. To test this hypothesis, we performed an EFA, following the methods established in Study 1. We predicted that, using the same guidelines we applied for conducting and interpreting EFA in Study 1, we would find support for a 4-factor solution, with factors that were similar to the factors revealed in that study (e.g., similar groups of capacities that loaded strongly on any given factor).

In line with our predictions, both parallel analysis and Weisman et al.'s factor retention criteria suggested a 4-factor solution, which accounted for 75% of the total variance in participants' capacity attributions. In this solution, all items loaded most strongly on the factors that they were selected to represent. Minimizing BIC suggested an 8-factor solution (accounting for 78% of the total variance).

Preregistered confirmatory analysis of perceived developmental trajectories We preregistered two hypotheses about the perceived developmental trajectories we anticipated to observe in Study 2. (1) We predicted that, on average, participants' perception of children's mental capacities would increase as a function of the target child's age, with participants generally attributing more and greater abilities to older children; and (2) We predicted that, in the domains of *cognition and control* and *social connection*, participants would perceive relatively more dramatic developmental changes extending later into childhood (i.e., lower intercepts and steeper slopes), whereas in the domains of *bodily sensation* and *negative affect* participants would perceive relatively smaller changes across development (i.e., higher intercepts and shallower slopes).

To test these hypotheses, we conducted a multilevel logistic regression via a generalized additive model, regressing item-level responses onto the domain (i.e., which of the four "factors" this capacity was

selected to represented) and a spline-based smooth of the target age of the child fitted separately for each domain, including the maximal random effects structure that would allow the model to converge. Using the "mgcv" package for R (Wood, 2017), we specified this model as follows: *gam(response ~ s(target age in years, by = domain) + domain, random = list(subject ID = ~1, capacity = ~1).* Attempting to model random slopes via generalized additive mixed models for domain or for target age resulted in pathological models (e.g., introducing systematic bias in predicted responses). Domain was dummy-coded with *cognition and control* as the baseline.

These tests deviated slightly from the preregistered analysis. We originally planned to use "factor scores" (which take into account participants' responses to all 20 items and the relations of those items to all 4 factors) rather than the summary scores described above as our dependent variable for these tests. We planned to derive these factor scores by projecting Study 2 data into the conceptual space defined by the factor solution for Study 1; however, this projection was impossible to implement, because Study 2 included only a subset of the variables used in Study 1. Furthermore, upon reflection, we decided that using any kind of factor scores (even factor scores derived only from Study 2 data) would be inappropriate for testing our hypotheses, which hinge on assessing the *absolute* degree to which participants attributed different aspects of mental life to children of different ages; factor scores, which function much like z-scores, are instead designed to capture individual differences in responses (i.e., the degree to which a given participant attributed relatively greater or lesser capacities compared to the average of all participants).

In addition, we note that we originally planned to use a multilevel linear regression to analyze these scores. However, we now consider it misguided to model participants' responses using gaussian distribution, because—both in principle and in our empirical dataset—capacity ratings behaved asymptotically, plateauing near the ceiling of the scale (100, labeled "completely capable") at some point in development. The approach described here—using the binomial distribution—is a far more accurate representation of these responses. Using generalized additive models via the "mgcv" package for R (Wood, 2017) allowed us to fine-tune the shape of these perceived developmental trajectories via spline-based smoothing (analogous to fitting polynomial terms in the context of linear regression, but without specifying in advance the order of the polynomials included in the model).

Study 3

Participants

301 US adults participated via MTurk in April 2019. Eligibility requirements were identical to Studies 1-2. Participants were paid \$2.90 for approximately 15-20 minutes of their time. As in Study 2, we recruited men and women separately.

According to self-report, participants ranged in age from 18-45 years (M = 31.67 years, sd = 5.78 years) and (per our recruitment strategy) included roughly equal numbers of men (53%) and women (47%). Participants predominantly identified as White (67%; <12% identified as any other race/ethnicity, identified as more than one race/ethnicity, or declined to disclose). 53% of participants had obtained at least a Bachelor's degree. 34% of participants indicated that they were parents.

An additional 79 participants completed the study but were excluded for failing two or more (out of eight) embedded attention checks or for failing to provide a reasonable response in well-formed English sentences to an open-ended comprehension check included at the end of the survey.

Materials and procedure

After completing a screening questionnaire (in which participants provided information about their gender/sex, age, and English abilities) and providing informed consent, participants were provided with an overview of the study, as follows: "In this study, we will ask you about the mental capacities of children of different ages. We will show you example photos of children at each of the following ages to remind you of

what children look like at that age, but please think about children of that age in general when answering these questions. We will ask you about 8 different mental capacities: feeling happy, getting hungry, controlling emotions, feeling distressed, feeling pain, reasoning about things, learning from other people, and feeling helpless. For each mental capacity, we will ask you to assess the extent to which newborns (at birth) have this mental capacity, then 4-day-olds, 1-month-olds, 2-month-olds, 4-month-olds, and so on, ending with 5-year-olds. We'll also ask for your opinion about the different factors that might influence the development of these capacities, such as the child being 'preprogrammed' to have certain abilities, or the child learning from the people or objects around him or her. At the end of the survey, we will ask you a few demographic questions about yourself. We are interested in your opinions about these questions. Please do not look up extra information about these questions—we just want to know what you think."

Participants then proceeded through eight trials, in which they assessed each of the eight capacities in turn; the order of capacities was randomized across participants. On each trial, participant assessed children at all 13 target ages used in Study 2 in a fixed order from youngest to oldest. Each age was presented along with a representative pair of photographs (also used in Studies 1 and 2), and participants were asked to answer the following question: "To what extent is a [newborn/4-day-old/1-month-old/etc.] capable of [this capacity]?" Participants responded on a sliding scale from 0 (labeled as "not at all capable") to 100 (labeled as "completely capable").

On each trial, at the bottom of the page participants completed a set of questions about the possible mechanisms that drive development for the capacity in question. First, they were asked, "In your opinion, how important is each of the following factors in the development of children's capacity for [capacity]?" They provided independent ratings on a Likert-type scale from 0 (labeled as "not at all important") to 6 (labeled as "extremely important"), for the following 10 developmental mechanisms: "the child is biologically 'preprogrammed' to have this ability"; "the child has experiences in the womb that give them this ability"; "the child's body grows and matures (for example, muscles get stronger, child gets taller)"; "the child's brain changes (for example, brain grows bigger, more or fewer connections between neurons)"; "the child's senses improve (for example, vision gets sharper, hearing improves)"; "the child observes the objects and the physical world around him or her"; "the child observes the people around him or her"; "the child interacts with the people around him or her"; "people explicitly teach the child how to do this"; "the child actively experiments with how to do this". These items were presented in a fixed order (roughly, from what we perceived to be the most innate or biological mechanisms of development, to what we perceived to be the most learned or social). They were then provided an opportunity to write in "any factors that play a role in the development of children's capacity for [capacity]". Finally, they were asked to choose among these 10 possible mechanisms (or to write in another mechanism) to answer the question, "If you had to choose just one, which of the following factors is the most important in the development of capacities for [capacity]?" On the final page of the survey, participants were asked to provide additional demographic information.

Capacities Because Study 3 included additional questions probing participants' theories of developmental mechanisms, we reduced the set of mental capacities from 20 in Study 2 to eight in the current study to maintain an appropriate level of participant burden. To select these eight capacities, we drew on the results of Study 2, selecting two items for each of the four factors identified by EFA. For each domain, we selected items that loaded strongly on the relevant factor, did not cross-load strongly on other factors, were sufficiently distinguishable from each other in meaning (i.e., not overly redundant), and captured our qualitative understanding of the domain that each factor corresponded to. We chose "controlling their emotions" and "reasoning about things" to represent the domain of *cognition and control*; "getting hungry" and "feeling pain" to represent the domain of *bodily sensation*; "learning from other people" and "feeling happy" to represent the domain of *social connection*; and "feeling distressed" and "feeling helpless" to represent the domain of *negative affect*. Capacities were presented in a random order for each participant.

Target ages and accompanying photographs The 13 target ages for this study were identical to Study 2, with the exception that we added the parenthetical phrase "(at birth)" when referring to newborns. Each target age was presented with the same pair of photographs used in Study 2. Target ages were presented in chronological order on each trial.

Analysis plan

Preregistered confirmatory analysis of perceived developmental trajectories We preregistered two hypotheses about the perceived developmental trajectories we anticipated to observe in Study 3; these were identical to our hypotheses for Study 2 (which were upheld in that study): (1) We predicted that, on average, participants' perception of children's mental capacities would increase as a function of the target child's age, with participants generally attributing more and greater abilities to older children; and (2) We predicted that, in the domains of *cognition and control* and *social connection*, participants would perceive relatively more dramatic developmental changes extending later into childhood (i.e., lower intercepts and steeper slopes), whereas in the domains of *bodily sensation* and *negative affect* participants would perceive relatively smaller changes across development (i.e., higher intercepts and shallower slopes).

Following Study 2, to test these hypotheses we conducted a multilevel logistic regression via a generalized additive model, regressing item-level responses onto the domain and a spline-based smooth of the target age of the child fitted separately for each domain, including the maximal random effects structure that would allow the model to converge. Using the "mgcv" package for R (Wood, 2017), we specified this model as follows: *gam(responses ~ s(target age in years, by = domain) + domain + s(subject ID, bs = "re") + s(capacity, bs = "re")*. Again, attempting to model random slopes for domain or for target age resulted in model convergence problems. Again, responses were rescaled to range from 0.005 to 0.995, and domain was dummy-coded with *cognition and control* as the baseline.

(We note that, in contrast to Study 2, in Study 3 we did preregister this analysis, although we preregistered a linear regression rather than a logistic regression via generalized additive model; see extended notes on our choice of this analysis approach under Study 2.)

Preregistered exploratory analysis of perceived developmental trajectories Beyond the two hypotheses described in the previous section, we also anticipated that perceptions of developmental trajectories in different domains would vary in their curvilinearity—e.g., development in one domain might be perceived to be gradual and linear, while development in another domain might be perceived to occur in stages, at an exponential rate, or to peak at a specific age. To explore these possibilities, we preregistered a regression analysis adding polynomial effects of target age; however, in our reconceptualization of the appropriate way to analyze our data given the distribution of the response variable (see Study 2), this exploratory analysis was superseded by the generalized additive model.

Preregistered exploratory analysis of developmental mechanisms Finally, we assessed which developmental mechanisms were considered to be the most or least important in the development of each of the individual mental capacities included in this study (*bodily sensation*, *negative affect*, *social connection*, and *cognition and control*). We specified in our preregistration that this assessment would be conducted primarily through visualization, but to complement this visualization we also conducted a multilevel linear regression regressing responses to this question onto developmental mechanism, domain, and an interaction between them, including the maximal random effects structure that allowed the model to converge. Using the "lme4" package for R (Bates et al., 2015), we specified this model as follows: *lmer(response ~ mechanism * domain + (1 + mechanism + domain | participant) + (1 | mechanism) + (1 | capacity).* We also conducted separate, analogous regression analyses for each mechanism separately. In all of these analyses, mechanism and domain were effect-coded for comparisons to the grand mean collapsing across mechanism types and domains.

Extended results

Study 1

As described in the main text, we began with an expansive exploration of US adults' attributions of mental life to infants and young children of different ages. We included a wide range of 60 capacities in this initial

exploration, from basic physiological sensations, to capacities for perception, cognition, and emotion, to abilities for self-regulation and social interaction. We asked participants to assess these capacities at three different ages (at birth, 9 months, and 5 years), with the goal of characterizing the conceptual structure underlying participants' reasoning about the development of mental life. Our primary analysis for this preregistered study was an exploratory factor analysis (EFA) of participants' capacity ratings. This analysis allowed us to examine which capacities tend to "hang together" in participants' assessments of the mental lives of infants and young children. Following previous work on mind perception, we argue that the suites of capacities revealed by EFA offer a meaningful approximation of the latent conceptual structure underlying participants' reasoning about the developing human mind.

In the main text, we presented the four-factor EFA solution suggested by parallel analysis. Here, we present extended results for this EFA and the alternative EFA solutions suggested by minimizing BIC and by Weisman et al.'s factor retention criteria (Weisman et al., 2017b), as well as a visualization of perceptions of development in these mental capacities.

Exploratory factor analyses (EFAs)

In order to determine how many factors to retain, we examined the results of three factor retention protocols:

(1) Parallel analysis (presented in the main text), which compares the observed correlation structure to the correlation structure arising from random datasets of the same size;
(2) Minimizing the Bayesian Information Criterion (BIC), which is one method of optimizing both goodness of fit and parsimony; and
(3) A set of factor retention criteria that have been used in Weisman et al.'s previous work (Weisman et al., 2017b), in which they retained factors with eigenvalues greater than 1.0, which individually accounted for greater than 5% of the shared variance before transformation or rotation, and which were the "dominant" factor (the factor with the strongest absolute factor loading) for at least one mental capacity after transformation. (In this protocol, a factor had to meet all three of these criteria in order to be retained).

Our interpretation of how best to characterize our dataset (i.e., how many factors we observe) was guided by the degree of consensus among these three protocols and the interpretability of the retained factors under each protocol. Here we present these three factor retention protocols separately.

All factor analyses were conducted using ordinary least squares to find the minimum residual solution via the "fa" function in the "psych" package for R (Revelle, 2021).

Parallel analysis As reported in the main text, parallel analysis suggested retaining 4 factors. We call these factors *cognition and control*, *social connection*, *bodily sensation*, and *negative affect*. Factor loadings, item complexity, and uniqueness are shown in Supplementary Table 1; see also Figure 1 (main text). The amount of total and shared variance accounted for by these factors is shown in Supplementary Table 2.

It is worth noting that a handful of items loaded relatively strongly on two factors (loadings $\geq$ 0.40), suggesting that these factors are not entirely distinct. The item "feeling distressed", for instance, loaded roughly equally strongly on both *bodily sensation* and *negative affect*—perhaps reflecting the idea that "distress" might be experienced both as a physical sensation (like pain) and an affective state (like frustration). Likewise, "seeing" loaded equally strongly on both *bodily sensation* and *social connection*—perhaps reflecting an understanding of vision as both a sensory ability and a critical part of many social interactions (eye contact). The relatively large degree of overlap between *cognition and control* and *social connection*, in particular, might indicate that participants believed these two constructs to be more strongly connected than other aspects of life; in fact, these two factors were the most strongly correlated of all pairs of factors ($\phi = 0.65$). (The next strongest correlation was between *bodily sensation* and *social connection*: $\phi = 0.59$. All other inter-factor correlations: $\phi \leq 0.50$.)

Table 1: Study 1, factor solution suggested by parallel analysis: Factor loadings, complexity, and uniqueness.

Capacity	Cog. Ctrl.	Soc. Conn.	Bod. Sens.	Neg. Afft.	Complexity	Uniqueness
planning	**1.01**	-0.11	-0.01	-0.04	1.03	0.14
having self control	**0.96**	-0.02	-0.01	-0.05	1.01	0.14
thinking before they act	**0.96**	-0.03	0.03	-0.04	1.01	0.15
having goals	**0.95**	-0.08	0.00	0.02	1.01	0.17
reasoning about things	**0.94**	0.00	-0.01	-0.05	1.01	0.16
controlling their emotions	**0.92**	-0.01	-0.02	-0.06	1.01	0.21
telling right from wrong	**0.91**	0.00	0.03	0.01	1.00	0.14
understanding what somebody else is thinking	**0.90**	-0.05	-0.04	0.03	1.01	0.22
focusing on a goal	**0.90**	0.02	0.00	-0.02	1.00	0.19
feeling guilty	**0.89**	-0.04	0.06	0.09	1.03	0.16
feeling embarrassed	**0.83**	0.04	0.01	0.09	1.03	0.17
feeling pride	**0.83**	0.07	-0.05	0.09	1.04	0.17
making choices	**0.69**	0.32	-0.04	-0.02	1.43	0.18
calming themselves down	**0.68**	0.25	-0.01	-0.05	1.27	0.30
detecting danger	**0.67**	0.07	0.10	0.12	1.14	0.32
feeling hopeless	**0.67**	0.01	0.01	0.28	1.34	0.28
remembering things	0.52	0.49	0.01	-0.10	2.07	0.23
imagining things	0.51	0.42	-0.01	0.03	1.94	0.26
recognizing others emotions	0.50	0.41	-0.04	0.03	1.95	0.32
feeling worried	0.42	0.24	0.05	0.35	2.64	0.25
getting hurt feelings	0.41	0.40	0.00	0.19	2.40	0.27
having wants and desires	0.29	0.26	0.17	0.27	3.62	0.40
feeling excited	0.00	**0.85**	-0.01	0.06	1.01	0.24
finding something funny	0.06	**0.84**	-0.02	0.00	1.01	0.25
loving somebody	0.05	**0.83**	-0.10	0.09	1.06	0.27
learning from other people	0.12	**0.80**	-0.02	0.01	1.05	0.24
feeling happy	-0.12	**0.79**	0.16	0.01	1.13	0.30
feeling loved	-0.05	**0.77**	0.04	0.07	1.03	0.35
recognizing somebody else	0.06	**0.76**	0.17	-0.10	1.14	0.27
getting pleasure from music	0.10	**0.62**	0.17	0.04	1.20	0.34
being afraid of somebody	0.02	**0.62**	0.11	0.26	1.41	0.26
listening to somebody	0.18	**0.61**	0.14	-0.03	1.29	0.36
having thoughts	0.26	0.59	0.10	0.02	1.45	0.29
feeling sad	0.00	0.58	0.15	0.24	1.50	0.31
feeling safe	0.02	0.54	0.24	0.13	1.51	0.37
feeling textures (for example, smooth, rough)	0.06	0.54	0.36	-0.05	1.81	0.34
getting angry	0.09	0.53	0.05	0.34	1.78	0.28
feeling pleasure	0.06	0.47	0.28	0.16	1.91	0.36
being angry at somebody	0.38	0.42	0.00	0.25	2.62	0.21
feeling lonely	0.16	0.42	0.10	0.35	2.38	0.31
feeling bored	0.32	0.42	0.04	0.27	2.67	0.25
feeling confused	0.16	0.41	0.10	0.36	2.42	0.29
feeling scared	-0.06	0.41	0.37	0.27	2.79	0.31
being aware of things	0.30	0.34	0.33	0.03	2.97	0.36
getting hungry	-0.06	-0.13	**0.90**	0.01	1.05	0.31
feeling pain	0.00	-0.07	**0.90**	0.03	1.01	0.25
feeling tired	0.00	-0.06	**0.88**	0.02	1.01	0.28
feeling thirsty	0.01	0.02	**0.84**	-0.04	1.01	0.28
feeling too hot or too cold	0.03	0.09	**0.77**	0.02	1.03	0.28
feeling physically uncomfortable	0.05	-0.02	**0.76**	0.18	1.12	0.29
hearing sounds	-0.04	0.21	**0.75**	-0.13	1.23	0.31
being comforted by physical touch	-0.03	0.17	**0.72**	-0.09	1.15	0.37
feeling distressed	0.07	0.00	0.49	0.45	2.03	0.34
seeing	0.01	0.46	0.47	-0.22	2.41	0.43
feeling calm	0.09	0.35	0.36	0.12	2.36	0.44

Table 1: Study 1, factor solution suggested by parallel analysis: Factor loadings, complexity, and uniqueness. *(continued)*

Capacity	Cog. Ctrl.	Soc. Conn.	Bod. Sens.	Neg. Afft.	Complexity	Uniqueness
feeling helpless	0.34	0.13	0.12	0.43	2.30	0.33
feeling overwhelmed	0.22	0.25	0.12	0.42	2.38	0.35
feeling frustrated	0.12	0.37	0.19	0.39	2.65	0.27
feeling annoyed	0.24	0.37	0.07	0.38	2.74	0.25
feeling neglected	0.16	0.26	0.25	0.36	3.16	0.36

Note:

Factor loadings with absolute values >0.60 are in bold.

Table 2: Study 1, factor solution suggested by parallel analysis: Variance accounted for.

	Cognition and control	Social connection	Bodily sensation	Negative affect
SS loadings	16.45	14.29	8.47	4.25
Proportion Var	0.27	0.24	0.14	0.07
Cumulative Var	0.27	0.51	0.65	0.72
Proportion Explained	0.38	0.33	0.19	0.10
Cumulative Proportion	0.38	0.71	0.90	1.00

Minimizing BIC Minimizing BIC suggested retaining 6 factors. (Note, however, that this solution does not converge.) Factor loadings, item complexity, and uniqueness are shown in Supplementary Table 3. The amount of total and shared variance accounted for by these factors is shown in Supplementary Table 4.

Table 3: Study 1, factor solution suggested by minimizing BIC: Factor loadings, complexity, and uniqueness.

Capacity	MR1	MR2	MR5	MR4	MR6	MR3	Complexity	Uniqueness
planning	**1.00**	-0.04	-0.05	0.02	0.00	-0.11	1.03	0.13
having self control	**0.96**	-0.01	0.01	-0.04	0.00	-0.04	1.01	0.14
thinking before they act	**0.96**	0.01	0.01	-0.02	0.00	-0.06	1.01	0.15
reasoning about things	**0.94**	-0.01	0.01	-0.03	0.02	-0.03	1.01	0.16
having goals	**0.94**	-0.05	0.00	0.08	-0.02	-0.10	1.04	0.16
controlling their emotions	**0.93**	0.00	0.06	-0.09	-0.04	-0.02	1.03	0.21
telling right from wrong	**0.91**	0.07	-0.04	-0.05	-0.02	0.11	1.05	0.14
focusing on a goal	**0.89**	-0.05	0.01	0.07	0.07	-0.11	1.06	0.18
understanding what somebody else is thinking	**0.89**	-0.04	-0.03	0.02	-0.02	0.02	1.01	0.22
feeling guilty	**0.87**	0.10	-0.04	-0.01	-0.08	0.17	1.12	0.14
feeling embarrassed	**0.82**	0.05	-0.04	0.00	-0.03	0.20	1.14	0.15
feeling pride	**0.82**	-0.05	0.03	0.07	0.00	0.08	1.04	0.17
calming themselves down	**0.71**	0.00	0.18	-0.02	0.08	-0.02	1.17	0.29
making choices	**0.70**	-0.04	0.06	0.04	0.22	0.07	1.24	0.17
detecting danger	**0.65**	0.08	-0.02	0.13	0.04	0.11	1.18	0.32
feeling hopeless	**0.64**	-0.02	0.09	0.22	-0.13	0.12	1.47	0.28
remembering things	0.56	0.04	0.16	-0.05	0.28	0.10	1.75	0.23
imagining things	0.53	0.04	0.17	0.00	0.15	0.19	1.67	0.25
recognizing others emotions	0.52	-0.04	0.16	0.08	0.19	0.09	1.64	0.32
getting hurt feelings	0.41	0.06	0.21	0.07	0.03	0.32	2.56	0.25
feeling worried	0.39	0.03	0.14	0.29	-0.03	0.26	3.02	0.25
having thoughts	0.30	0.10	0.27	0.09	0.27	0.09	3.65	0.28
getting hungry	-0.05	**0.94**	0.01	-0.09	-0.16	0.08	1.10	0.24
feeling pain	-0.01	**0.86**	-0.02	0.05	-0.02	0.03	1.01	0.24
feeling thirsty	0.01	**0.85**	-0.01	-0.03	0.03	0.06	1.02	0.26

Table 3: Study 1, factor solution suggested by minimizing BIC: Factor loadings, complexity, and uniqueness. *(continued)*

Capacity	MR1	MR2	MR5	MR4	MR6	MR3	Complexity	Uniqueness
feeling tired	0.00	**0.83**	0.05	0.05	-0.05	-0.02	1.02	0.27
hearing sounds	-0.02	**0.72**	-0.01	0.00	0.25	0.00	1.24	0.30
feeling too hot or too cold	0.03	**0.68**	0.02	0.18	0.14	-0.07	1.25	0.28
being comforted by physical touch	0.01	**0.66**	0.29	0.01	0.03	-0.20	1.60	0.34
feeling physically uncomfortable	0.01	**0.61**	-0.08	0.37	0.11	-0.06	1.79	0.27
seeing	0.06	0.49	0.06	-0.10	0.39	0.04	2.09	0.41
feeling loved	0.02	0.06	**0.89**	-0.03	-0.04	0.01	1.02	0.19
loving somebody	0.12	-0.06	**0.66**	0.02	0.11	0.16	1.25	0.23
feeling safe	0.05	0.17	0.57	0.21	0.06	-0.09	1.57	0.31
feeling happy	-0.06	0.13	0.50	0.13	0.30	0.01	2.01	0.29
feeling sad	0.02	0.16	0.39	0.20	0.07	0.24	2.71	0.30
feeling excited	0.05	0.01	0.38	0.11	0.35	0.20	2.77	0.24
feeling lonely	0.15	0.04	0.38	0.35	-0.02	0.14	2.65	0.29
getting pleasure from music	0.13	0.12	0.33	0.17	0.29	0.02	3.16	0.32
feeling calm	0.10	0.25	0.33	0.27	0.11	-0.11	3.65	0.40
feeling distressed	0.00	0.29	0.01	**0.65**	0.00	0.00	1.39	0.30
feeling overwhelmed	0.17	-0.01	0.14	0.54	0.06	0.10	1.44	0.33
feeling helpless	0.30	0.03	0.20	0.44	-0.11	0.11	2.52	0.33
feeling confused	0.13	0.02	0.09	0.44	0.19	0.24	2.28	0.28
feeling frustrated	0.10	0.11	0.17	0.44	0.09	0.22	2.23	0.26
feeling neglected	0.14	0.16	0.35	0.38	-0.09	0.06	2.84	0.33
feeling scared	-0.07	0.31	0.17	0.33	0.15	0.19	3.69	0.31
having wants and desires	0.27	0.10	0.13	0.33	0.07	0.12	2.93	0.40
feeling pleasure	0.06	0.18	0.28	0.32	0.22	-0.02	3.50	0.33
feeling textures (for example, smooth, rough)	0.08	0.31	0.15	0.13	0.38	0.01	2.65	0.32
learning from other people	0.17	0.00	0.31	0.09	0.38	0.17	2.94	0.23
recognizing somebody else	0.12	0.22	0.29	-0.05	0.38	0.18	3.37	0.26
finding something funny	0.11	0.06	0.29	-0.02	0.37	0.33	3.19	0.22
being aware of things	0.30	0.24	-0.02	0.23	0.36	0.01	3.58	0.32
listening to somebody	0.21	0.14	0.19	0.07	0.35	0.12	3.05	0.36
being angry at somebody	0.37	0.05	0.10	0.16	0.10	0.40	2.63	0.17
getting angry	0.08	0.08	0.23	0.25	0.09	0.40	2.71	0.25
feeling annoyed	0.21	0.06	0.09	0.35	0.08	0.37	2.87	0.23
feeling bored	0.31	0.07	0.11	0.20	0.11	0.36	3.13	0.22
being afraid of somebody	0.02	0.13	0.26	0.24	0.19	0.32	3.89	0.24

Note:
Factor loadings with absolute values >0.60 are in bold.

Table 4: Study 1, factor solution suggested by minimizing BIC: Variance accounted for.

	MR1	MR2	MR5	MR4	MR6	MR3
SS loadings	16.56	7.47	7.04	5.69	4.08	3.67
Proportion Var	0.28	0.12	0.12	0.09	0.07	0.06
Cumulative Var	0.28	0.40	0.52	0.61	0.68	0.74
Proportion Explained	0.37	0.17	0.16	0.13	0.09	0.08
Cumulative Proportion	0.37	0.54	0.70	0.83	0.92	1.00

We consider the first of these factors (“MR1”) to resonate with the construct we have called *cognition and control*, the second (“MR2”) to resonate with the construct we have called *bodily sensation*, the third (“MR5”) to resonate with the construct we have called *social connection*, and the fourth (“MR4”) to resonate with the construct we have called *negative affect*. The fifth factor (“MR6”) appears to pick out capacities particularly relevant to social interaction (e.g., social learning, person recognition, humor, listening), and the

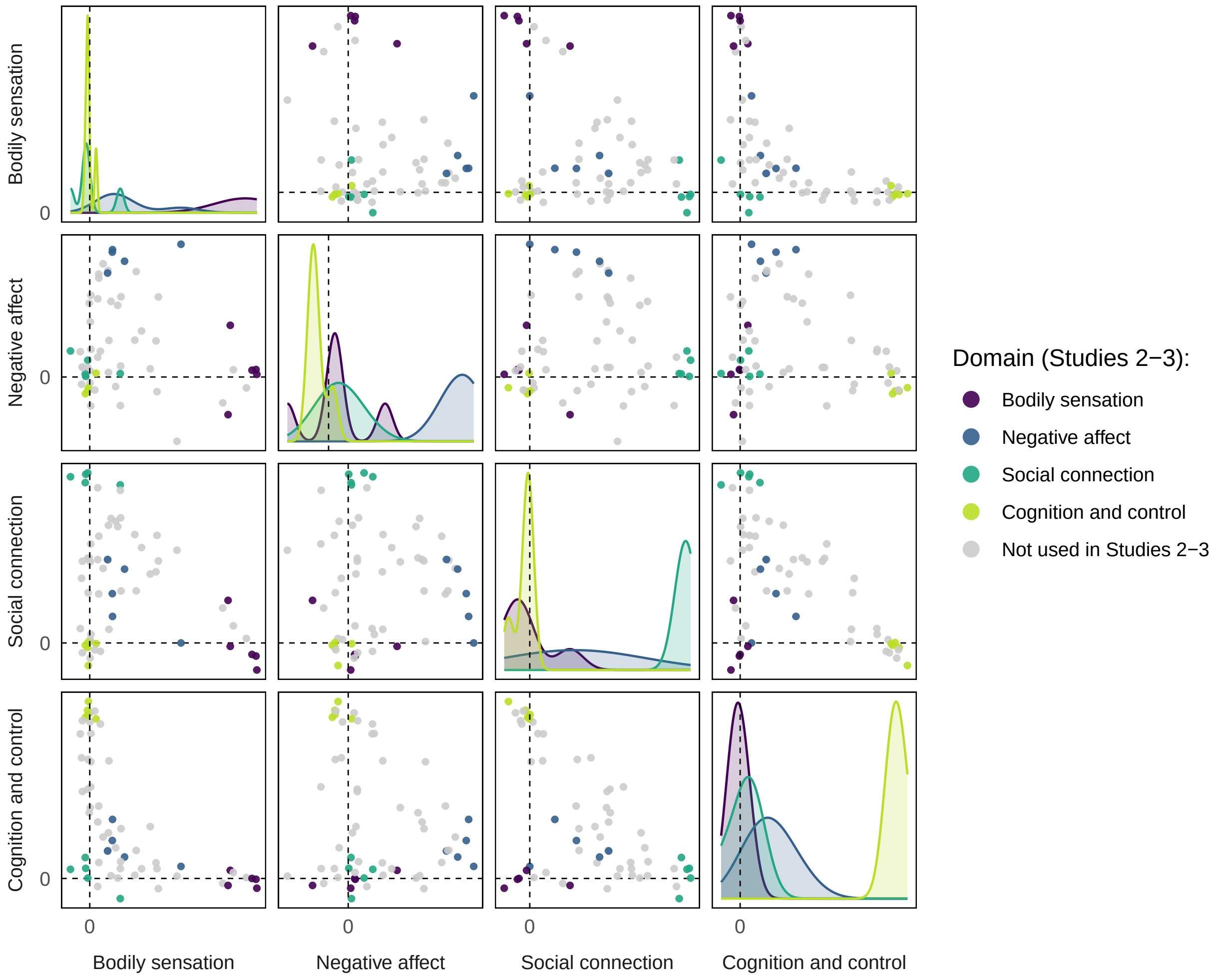

Figure S1: Study 1, scatterplot matrix ('pairs plot') visualizing the relationships among factor loadings and the distributions of factor loadings across items for the four-factor solution suggested by parallel analysis (loadings presented in Figure 1). Along the diagonal are density plots showing, for each of the four factors, the distribution of factor loadings for the subset of capacities later used to represent each 'domain' in Studies 2-3. Panels in the upper and lower triangles are scatterplots of factor loadings on each pair of factors, including all of the capacities included in Study 1.

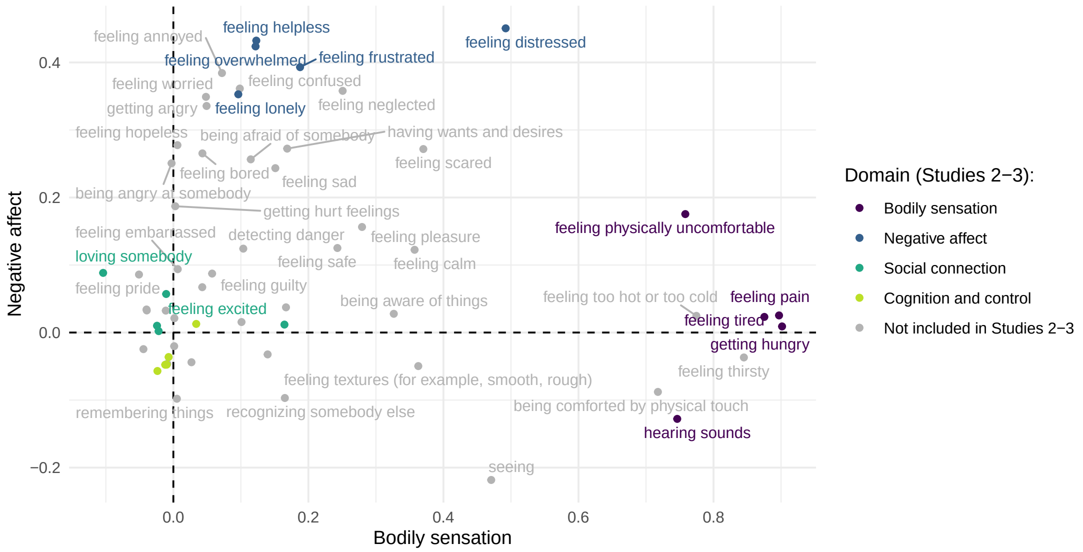

Figure S2: Study 1, scatter plot visualizing the relationship between factor loadings on the factors we have labeled 'bodily sensation' and 'negative affect' in the factor solution suggested by parallel analysis (loadings presented in Figure 1). The subset of capacities used in Studies 2-3 are color-coded according to the domain they were selected to represent in those studies.

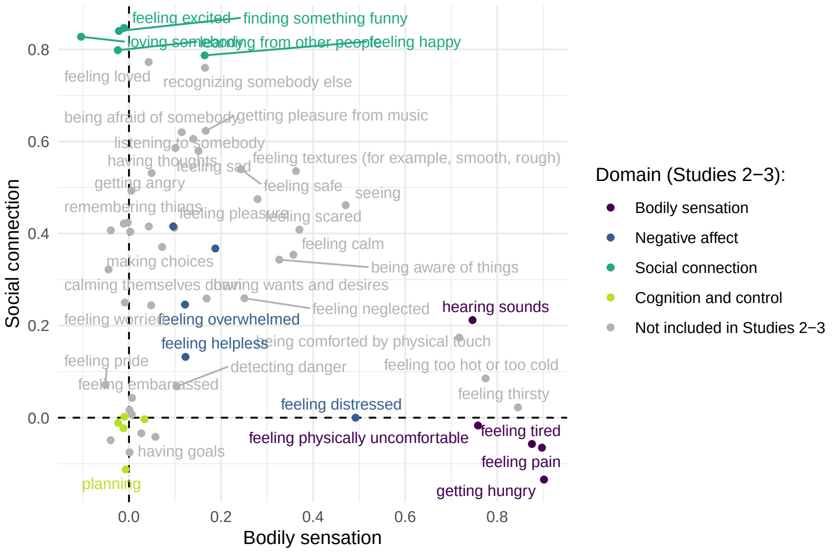

Figure S3: Study 1, scatter plot visualizing the relationship between factor loadings on the factors we have labeled 'bodily sensation' and 'social connection' in the factor solution suggested by parallel analysis (loadings presented in Figure 1). The subset of capacities used in Studies 2-3 are color-coded according to the domain they were selected to represent in those studies.

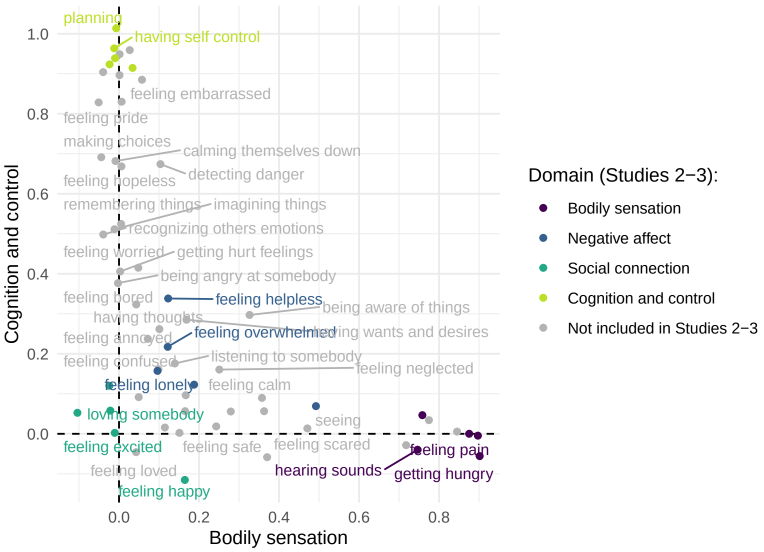

Figure S4: Study 1, scatter plot visualizing the relationship between factor loadings on the factors we have labeled ‘bodily sensation’ and ‘cognition and control’ in the factor solution suggested by parallel analysis (loadings presented in Figure 1). The subset of capacities used in Studies 2-3 are color-coded according to the domain they were selected to represent in those studies.

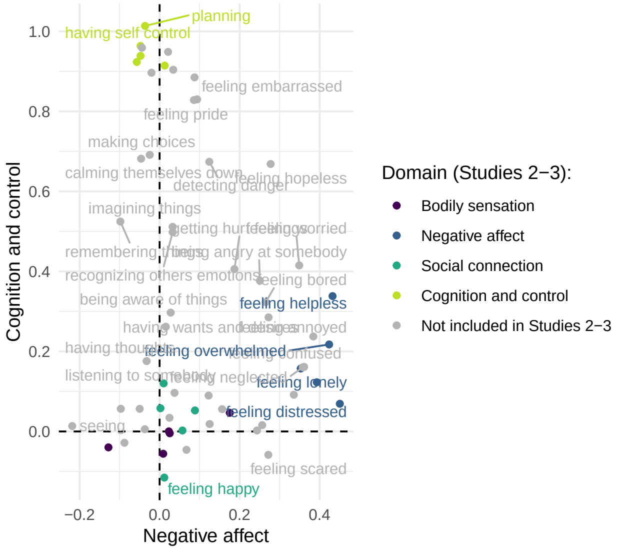

Figure S5: Study 1, scatter plot visualizing the relationship between factor loadings on the factors we have labeled ‘negative affect’ and ‘social connection’ in the factor solution suggested by parallel analysis (loadings presented in Figure 1). The subset of capacities used in Studies 2-3 are color-coded according to the domain they were selected to represent in those studies.

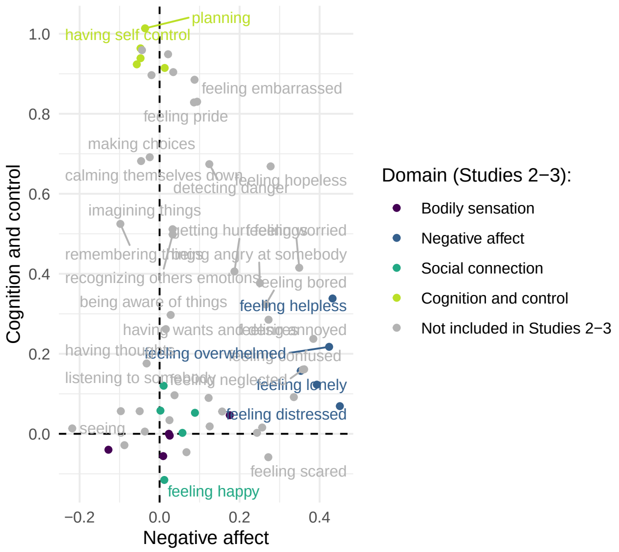

Figure S6: Study 1, scatter plot visualizing the relationship between factor loadings on the factors we have labeled 'negative affect' and 'cognition and control' in the factor solution suggested by parallel analysis (loadings presented in Figure 1). The subset of capacities used in Studies 2-3 are color-coded according to the domain they were selected to represent in those studies.

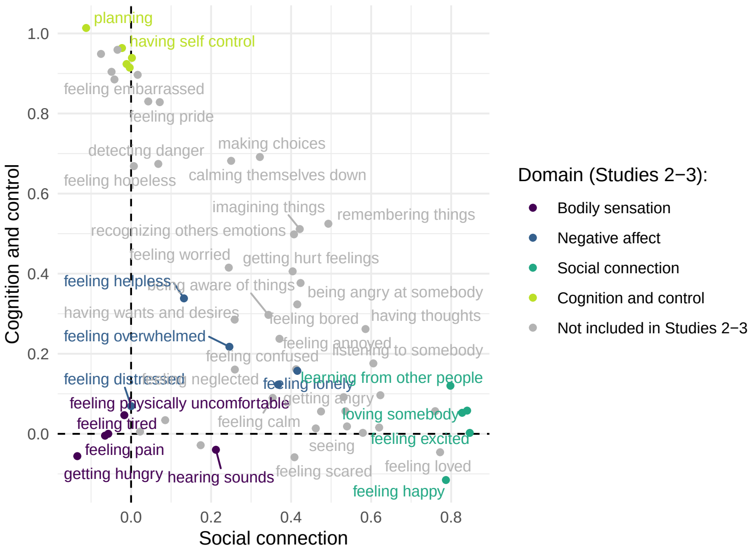

Figure S7: Study 1, scatter plot visualizing the relationship between factor loadings on the factors we have labeled 'social connection' and 'cognition and control' in the factor solution suggested by parallel analysis (loadings presented in Figure 1). The subset of capacities used in Studies 2-3 are color-coded according to the domain they were selected to represent in those studies.

sixth factor ("MR3") appears to pick out negative emotions (e.g., anger, annoyance, boredom, social fear); however, there were no capacities that loaded strongly on either of these capacities.

Weisman et al.'s (2017) factor retention criteria Weisman et al.'s factor retention criteria suggested retaining 2 factors. Factor loadings, item complexity, and uniqueness are shown in Supplementary Table 5. The amount of total and shared variance accounted for by these factors is shown in Supplementary Table 6.

Table 5: Study 1, factor solution suggested by Weisman et al.'s (2017) factor retention criteria.

Capacity	MR1	MR2	Complexity	Uniqueness
planning	**0.97**	-0.18	1.07	0.18
having self control	**0.96**	-0.13	1.04	0.18
reasoning about things	**0.94**	-0.11	1.03	0.19
having goals	**0.94**	-0.12	1.03	0.20
thinking before they act	**0.94**	-0.10	1.02	0.19
telling right from wrong	**0.93**	-0.04	1.00	0.17
feeling pride	**0.93**	-0.05	1.01	0.18
understanding what somebody else is thinking	**0.93**	-0.14	1.05	0.24
controlling their emotions	**0.92**	-0.13	1.04	0.24
focusing on a goal	**0.92**	-0.08	1.01	0.22
feeling guilty	**0.91**	-0.01	1.00	0.19
feeling embarrassed	**0.91**	-0.01	1.00	0.18
making choices	**0.86**	0.09	1.02	0.19
feeling hopeless	**0.80**	0.05	1.01	0.32
calming themselves down	**0.80**	0.07	1.02	0.31
detecting danger	**0.75**	0.13	1.06	0.34
imagining things	**0.74**	0.22	1.18	0.27
remembering things	**0.72**	0.23	1.20	0.27
recognizing others emotions	**0.72**	0.18	1.13	0.33
being angry at somebody	**0.69**	0.32	1.42	0.23
getting hurt feelings	**0.68**	0.29	1.35	0.28
feeling worried	**0.67**	0.29	1.37	0.29
feeling bored	**0.62**	0.37	1.64	0.27
feeling annoyed	0.56	0.43	1.87	0.30
feeling helpless	0.56	0.34	1.65	0.41
having thoughts	0.52	0.45	1.96	0.31
learning from other people	0.51	0.47	1.99	0.31
loving somebody	0.50	0.44	1.97	0.36
having wants and desires	0.48	0.41	1.95	0.42
feeling confused	0.48	0.48	2.00	0.33
feeling overwhelmed	0.48	0.42	1.96	0.42
feeling lonely	0.48	0.48	2.00	0.35
feeling pain	-0.24	**0.88**	1.14	0.35
feeling tired	-0.22	**0.87**	1.13	0.37
feeling thirsty	-0.20	**0.86**	1.11	0.37
hearing sounds	-0.17	**0.85**	1.08	0.37
feeling too hot or too cold	-0.10	**0.85**	1.03	0.34
getting hungry	-0.33	**0.84**	1.30	0.43
feeling physically uncomfortable	-0.07	**0.82**	1.01	0.37
being comforted by physical touch	-0.16	**0.81**	1.07	0.42
feeling scared	0.15	**0.74**	1.09	0.32
seeing	0.03	**0.69**	1.00	0.51
feeling textures (for example, smooth, rough)	0.20	**0.69**	1.17	0.37
feeling happy	0.22	**0.68**	1.20	0.37
feeling distressed	0.14	**0.66**	1.09	0.46
feeling pleasure	0.28	**0.64**	1.36	0.36
feeling safe	0.26	**0.63**	1.34	0.38

Table 5: Study 1, factor solution suggested by Weisman et al.'s (2017) factor retention criteria. *(continued)*

Capacity	MR1	MR2	Complexity	Uniqueness
feeling calm	0.22	**0.63**	1.24	0.43
feeling sad	0.34	**0.61**	1.56	0.33
recognizing somebody else	0.33	**0.60**	1.56	0.34
being afraid of somebody	0.38	**0.60**	1.70	0.29
getting pleasure from music	0.37	0.57	1.71	0.36
feeling loved	0.33	0.56	1.63	0.42
feeling frustrated	0.41	0.56	1.85	0.32
feeling excited	0.42	0.54	1.89	0.33
feeling neglected	0.37	0.54	1.78	0.40
being aware of things	0.40	0.54	1.84	0.37
finding something funny	0.46	0.50	1.99	0.34
getting angry	0.47	0.50	1.99	0.32
listening to somebody	0.42	0.50	1.94	0.40

Note:
Factor loadings with absolute values >0.60 are in bold.

Table 6: Study 1, factor solution suggested by Weisman et al.'s (2017) factor retention criteria: Variance accounted for.

	MR1	MR2
SS loadings	23.33	17.58
Proportion Var	0.39	0.29
Cumulative Var	0.39	0.68
Proportion Explained	0.57	0.43
Cumulative Proportion	0.57	1.00

We consider the first of these factors ("MR1") to resonate most strongly with the construct we have called *cognition and control*, and the second ("MR2") to resonate most strongly with the construct we have called *bodily sensation*. These factors are also quite similar to the constructs of "agency" and "experience" in Gray et al.'s original studies of mind perception (Gray et al., 2007).

Four-factor solution, varimax rotation Throughout this manuscript we present EFA results after oblique ("oblimin") transformation. In recent years, oblique transformation has become the preferred approach for many researchers because it allows factors to be correlated with each others (to the extent that these correlations are suggested by the data), thus imposing fewer restrictions on the types of factor structures the might be surfaced by the analysis.

Here we present one alternative approach, using varimax rotation rather than oblique transformation. This yields a set of orthogonal (uncorrelated) factors.

This rotation is very similar to the oblique transformation that is the focus of our interpretation in the main text. We consider the first of these factors ("MR3") to resonate with the construct we have called *social connection*, the second ("MR2") to resonate with the construct we have called *bodily sensation*, the third ("MR1") to resonate with the construct we have called *cognition and control*, and the fourth ("MR4") to resonate with the construct we have called *negative affect*. We do note, however, that in this rotation there were no items that loaded most strongly on this fourth factor, in line with the idea that our results provide only moderate evidence for *negative affect* as a fourth, distinct, domain in participants' representations of the development of mental life.

Factor loadings, item complexity, and uniqueness are shown in Supplementary Table 7. The amount of total and shared variance accounted for by these factors is shown in Supplementary Table 8.

Table 7: Study 1, four-factor solution after varimax rotation: Factor loadings, complexity, and uniqueness.

Capacity	MR3	MR2	MR1	MR4	Complexity	Uniqueness
planning	**0.91**	0.03	0.14	0.06	1.06	0.14
having self control	**0.90**	0.05	0.20	0.05	1.11	0.14
thinking before they act	**0.89**	0.09	0.20	0.06	1.13	0.15
having goals	**0.89**	0.06	0.17	0.11	1.11	0.17
reasoning about things	**0.89**	0.06	0.21	0.05	1.14	0.16
telling right from wrong	**0.88**	0.12	0.23	0.11	1.20	0.14
feeling guilty	**0.87**	0.14	0.21	0.17	1.25	0.16
controlling their emotions	**0.86**	0.04	0.20	0.04	1.11	0.21
focusing on a goal	**0.86**	0.08	0.22	0.08	1.17	0.19
understanding what somebody else is thinking	**0.86**	0.03	0.17	0.11	1.12	0.22
feeling pride	**0.85**	0.07	0.26	0.17	1.28	0.17
feeling embarrassed	**0.85**	0.12	0.25	0.18	1.31	0.17
making choices	**0.79**	0.14	0.41	0.08	1.60	0.18
calming themselves down	**0.75**	0.14	0.35	0.06	1.52	0.30
feeling hopeless	**0.74**	0.13	0.22	0.32	1.63	0.28
detecting danger	**0.72**	0.22	0.26	0.20	1.63	0.32
imagining things	0.68	0.21	0.46	0.13	2.08	0.26
remembering things	0.68	0.23	0.50	0.02	2.10	0.23
recognizing others emotions	0.66	0.18	0.44	0.12	2.00	0.32
being angry at somebody	0.63	0.26	0.48	0.31	2.79	0.21
getting hurt feelings	0.63	0.24	0.46	0.26	2.56	0.27
feeling worried	0.62	0.26	0.37	0.39	2.81	0.25
feeling bored	0.58	**0.30**	0.47	0.32	3.11	0.25
feeling helpless	0.53	**0.30**	0.30	0.45	3.21	0.33
feeling annoyed	0.52	**0.33**	0.44	0.42	3.64	0.25
having wants and desires	0.48	**0.36**	0.36	0.32	3.58	0.40
feeling overwhelmed	0.46	**0.33**	0.36	0.44	3.73	0.35
feeling pain	0.01	**0.85**	0.14	0.11	1.09	0.25
feeling tired	0.02	**0.83**	0.14	0.10	1.09	0.28
feeling thirsty	0.04	**0.82**	0.19	0.06	1.12	0.28
getting hungry	-0.07	**0.82**	0.07	0.08	1.05	0.31
feeling too hot or too cold	0.11	**0.79**	0.25	0.12	1.28	0.28
hearing sounds	0.04	0.78	0.29	-0.02	1.29	0.31
feeling physically uncomfortable	0.13	0.77	0.19	0.24	1.38	0.29
being comforted by physical touch	0.05	0.74	0.27	0.01	1.27	0.37
seeing	0.15	0.59	0.43	-0.09	2.04	0.43
feeling distressed	0.24	0.59	0.21	0.46	2.56	0.34
feeling scared	0.23	0.58	0.45	0.32	2.86	0.31
feeling textures (for example, smooth, rough)	0.28	0.56	0.51	0.06	2.50	0.34
feeling calm	0.29	0.52	0.41	0.20	2.85	0.44
feeling pleasure	0.32	0.50	0.48	0.23	3.12	0.36
being aware of things	0.45	0.49	0.42	0.13	3.11	0.36
feeling neglected	0.40	0.44	0.37	0.39	3.92	0.36
feeling excited	0.38	0.34	0.69	0.15	2.21	0.24
finding something funny	0.41	0.32	**0.68**	0.10	2.19	0.25
loving somebody	0.43	0.26	**0.67**	0.17	2.19	0.27
learning from other people	0.46	0.31	**0.67**	0.11	2.32	0.24
feeling happy	0.24	0.47	**0.64**	0.11	2.20	0.30
recognizing somebody else	0.35	0.45	**0.64**	0.02	2.41	0.27
feeling loved	0.31	0.36	**0.63**	0.15	2.26	0.35
being afraid of somebody	0.37	0.42	**0.58**	0.31	3.27	0.26
getting pleasure from music	0.38	0.43	**0.57**	0.13	2.81	0.34
having thoughts	0.51	0.36	0.56	0.12	2.82	0.29
listening to somebody	0.42	0.39	0.55	0.07	2.77	0.36
feeling sad	0.34	0.43	0.54	0.30	3.29	0.31

Table 7: Study 1, four-factor solution after varimax rotation: Factor loadings, complexity, and uniqueness. *(continued)*

Capacity	MR3	MR2	MR1	MR4	Complexity	Uniqueness
getting angry	0.43	0.35	0.52	0.37	3.61	0.28
feeling safe	0.30	0.48	0.51	0.20	2.95	0.37
feeling confused	0.46	0.36	0.46	0.40	3.84	0.29
feeling lonely	0.45	0.35	0.46	0.39	3.83	0.31
feeling frustrated	0.42	0.43	0.44	0.42	3.99	0.27

Note:
Factor loadings with absolute values >0.60 are in bold.

Table 8: Study 1, four-factor solution after varimax rotation: Variance accounted for.

	Cognition and control	Bodily sensation	Social connection	Negative affect
SS loadings	18.87	11.03	10.46	3.11
Proportion Var	0.31	0.18	0.17	0.05
Cumulative Var	0.31	0.50	0.67	0.72
Proportion Explained	0.43	0.25	0.24	0.07
Cumulative Proportion	0.43	0.69	0.93	1.00

Capacity ratings: Developmental trajectories

Study 1 also offered a first glimpse into US adults' perceptions of the development of mental life. Supplementary Figure 8 provides a visualization of mean "scores" for each domain of mental life. To calculate these scores, we sorted each of the 60 mental capacities included in this study into on of the four domains (*bodily sensation*, *negative affect*, *social connection* or *cognition and control*) according to the factor on which it loaded the most strongly and positively (see Supplementary Table 1); for each participant, we then took the average of their responses to capacities in each domain at each target age. As we go on to confirm in Studies 2-3, this visualization suggests that participants perceived capacities for *bodily sensation* to be substantial at birth and to develop relatively little over the first five years of life; in contrast, they perceived capacities for *negative affect*, *social connection*, and in particular *cognition and control* to be much more limited at birth and to development dramatically over the first five years of life.

Study 2

As described in the main text, in Study 2 we replicated the conceptual structure identified by Study 1 and then used it to chart how different aspects of mental life are perceived to change over development. The design of Study 2 was nearly identical to Study 1, except that instead of assessing 60 capacities for 3 target ages, each participant assessed 20 capacities for 13 target ages.

In the main text, we briefly described the four-factor EFA solution suggested by parallel analysis, and presented the results of a generalized additive model (GAM) predicting participants' responses as a function of target age, domain, and interactions between them. Here, we present the full results for the EFA mentioned in the main text, an alternative EFA solutions suggested by minimizing BIC, and the full results of the GAM.

Exploratory factor analyses (EFAs)

As in Study 1, we examined the results of three factor retention protocols (parallel analysis, presented in the main text; minimizing BIC; and Weisman et al.'s factor retention criteria (Weisman et al., 2017b)), and our interpretation of how best to characterize our dataset was guided by the degree of consensus among these

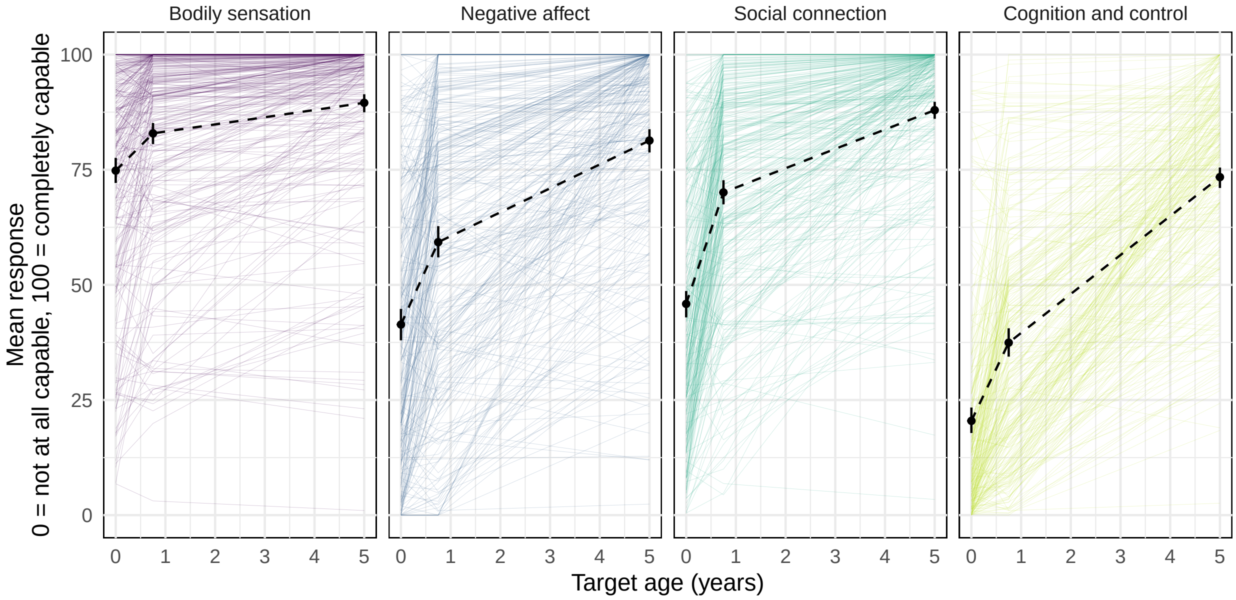

Figure S8: Perceived developmental trajectories for four domains of mental life (Study 1). Lighter lines represent individual participants' scores, black points correspond to mean scores across the sample, and error bars are bootstrapped 95% confidence intervals.

three protocols and the interpretability of the retained factors under each protocol. Here we present these three factor retention protocols separately.

Parallel analysis As reported in the main text, parallel analysis suggested retaining 4 factors, and these factors were very similar to those found in Study 1 (*bodily sensation*, *negative affect*, *social connection*, and *cognition and control*); each item loaded most strongly on the factor it was selected to represent. Factor loadings, item complexity, and uniqueness are shown in Supplementary Table 9; see also Figure 1 (main text). The amount of total and shared variance accounted for by these factors is shown in Supplementary Table 10.

Table 9: Study 2, factor solution suggested by parallel analysis: Factor loadings, complexity, and uniqueness.

Capacity	Cog. Ctrl.	Neg. Afft.	Soc. Conn.	Bod. Sens.	Complexity	Uniqueness
having self control	**0.96**	-0.02	-0.01	0.01	1.00	0.11
controlling their emotions	**0.94**	-0.04	0.01	-0.01	1.00	0.15
telling right from wrong	**0.93**	-0.03	0.04	0.00	1.01	0.12
planning	**0.90**	0.07	-0.05	0.00	1.02	0.18
reasoning about things	**0.89**	0.05	0.02	0.00	1.01	0.14
feeling overwhelmed	0.02	**0.84**	0.10	-0.06	1.04	0.23
feeling distressed	-0.01	**0.81**	-0.09	0.19	1.14	0.25
feeling frustrated	0.03	**0.80**	0.08	0.03	1.03	0.22
feeling helpless	0.08	**0.77**	0.09	-0.05	1.06	0.30
feeling lonely	0.05	**0.60**	0.27	0.03	1.41	0.30
feeling happy	-0.07	0.05	**0.85**	0.09	1.05	0.22
finding something funny	0.09	-0.01	**0.83**	0.02	1.03	0.22
feeling excited	-0.01	0.16	**0.79**	-0.01	1.09	0.22
loving somebody	0.15	0.07	**0.66**	0.01	1.13	0.37
learning from other people	0.31	0.03	0.48	0.05	1.74	0.45
getting hungry	-0.01	-0.06	0.02	**0.88**	1.01	0.27

Table 9: Study 2, factor solution suggested by parallel analysis: Factor loadings, complexity, and uniqueness. *(continued)*

Capacity	Cog. Ctrl.	Neg. Afft.	Soc. Conn.	Bod. Sens.	Complexity	Uniqueness
feeling pain	0.03	0.04	-0.01	**0.86**	1.01	0.22
feeling tired	-0.01	0.16	0.02	**0.72**	1.10	0.31
hearing sounds	0.01	-0.12	0.29	**0.67**	1.45	0.43
feeling physically uncomfortable	0.02	0.41	-0.12	0.56	1.95	0.36

Note:
Factor loadings with absolute values >0.60 are in bold.

Table 10: Study 2, factor solution suggested by parallel analysis: Variance accounted for.

	Cognition and control	Negative affect	Social connection	Bodily sensation
SS loadings	4.64	3.74	3.42	3.15
Proportion Var	0.23	0.19	0.17	0.16
Cumulative Var	0.23	0.42	0.59	0.75
Proportion Explained	0.31	0.25	0.23	0.21
Cumulative Proportion	0.31	0.56	0.79	1.00

Minimizing BIC Minimizing BIC suggested retaining 8 factors. (Note, however, that this solution does not converge.) Factor loadings, item complexity, and uniqueness are shown in Supplementary Table 11. The amount of total and shared variance accounted for by these factors is shown in Supplementary Table 12.

Table 11: Study 2, factor solution suggested by minimizing BIC: Factor loadings, complexity, and uniqueness.

Capacity	MR2	MR3	MR4	MR1	MR5	MR6	MR7	MR8	Complexity	Uniqueness
having self control	**0.98**	0.02	0.01	0.00	-0.01	-0.04	-0.04	-0.01	1.01	0.10
controlling their emotions	**0.96**	0.01	0.03	-0.03	0.00	-0.06	-0.04	-0.01	1.02	0.14
telling right from wrong	**0.92**	0.00	0.01	0.00	-0.02	0.04	-0.01	0.00	1.01	0.12
planning	**0.88**	-0.03	-0.04	0.04	0.03	0.03	0.05	0.00	1.02	0.18
reasoning about things	**0.86**	-0.02	-0.01	0.00	0.04	0.08	0.08	0.02	1.04	0.14
getting hungry	-0.01	**0.94**	-0.05	-0.02	-0.01	0.02	-0.05	0.04	1.02	0.23
feeling pain	0.02	**0.76**	0.02	-0.02	0.10	-0.04	0.12	-0.08	1.12	0.22
feeling tired	0.01	**0.71**	0.02	0.19	0.02	-0.02	-0.03	-0.02	1.16	0.30
hearing sounds	0.00	**0.63**	0.24	-0.06	-0.04	0.08	0.06	-0.02	1.37	0.43
feeling physically uncomfortable	-0.01	0.38	0.01	0.30	0.08	0.00	0.30	-0.08	3.07	0.30
feeling excited	0.04	0.00	**0.90**	0.04	0.02	-0.05	0.02	0.06	1.03	0.14
feeling happy	-0.03	0.05	**0.76**	0.06	0.06	0.07	-0.05	-0.12	1.12	0.20
finding something funny	0.08	0.03	**0.63**	-0.05	0.06	0.23	0.03	0.02	1.35	0.23
feeling distressed	0.02	0.05	-0.01	**0.84**	0.02	0.02	0.04	-0.11	1.05	0.18
feeling frustrated	0.06	0.05	0.17	**0.66**	0.07	-0.01	0.01	0.12	1.27	0.20
feeling overwhelmed	0.02	0.00	0.09	0.57	0.21	0.08	0.01	0.19	1.62	0.22
feeling lonely	0.01	0.02	0.04	-0.02	**0.88**	0.02	0.00	-0.07	1.02	0.17
feeling helpless	0.06	0.04	0.01	0.17	**0.63**	-0.01	0.04	0.18	1.36	0.25
learning from other people	0.19	0.04	0.11	0.08	0.03	0.52	0.06	0.03	1.49	0.36
loving somebody	0.10	0.05	0.21	0.07	0.22	0.40	-0.18	-0.08	3.11	0.30

Note:
Factor loadings with absolute values >0.60 are in bold.

Table 12: Study 2, factor solution suggested by minimizing BIC: Variance accounted for.

	MR2	MR3	MR4	MR1	MR5	MR6	MR7	MR8
SS loadings	4.55	2.91	2.55	2.29	1.90	0.94	0.28	0.18
Proportion Var	0.23	0.15	0.13	0.11	0.09	0.05	0.01	0.01
Cumulative Var	0.23	0.37	0.50	0.61	0.71	0.76	0.77	0.78
Proportion Explained	0.29	0.19	0.16	0.15	0.12	0.06	0.02	0.01
Cumulative Proportion	0.29	0.48	0.64	0.79	0.91	0.97	0.99	1.00

We consider the first of these factors ("MR2") to resonate with the construct we have called *cognition and control*, the second ("MR3") to resonate with the construct we have called *bodily sensation*, the third ("MR4") to resonate with the construct we have called *social connection*, and the fourth and fifth ("MR1" and "MR5") to resonate with the construct we have called *negative affect*. The sixth factor ("MR6") appears to pick out capacities particularly relevant to social interaction (e.g., social learning, love). We have no strong intuitions about how to interpret the seventh and eighth factors ("MR7" and "MR8").

Weisman et al.'s (2017) factor retention criteria Weisman et al.'s factor retention criteria suggested retaining 4 factors—i.e., the same 4-factor solution suggested by parallel analysis. (See Supplementary Tables 9 and 10.)

Capacity ratings: Developmental trajectories

Our primary goal in Study 2 was to chart perceptions of the development of mental life over the first five years. As described in the main text, we preregistered two hypotheses about the perceived developmental trajectories we anticipated to observe in Study 2:

(1) We predicted that, on average, participants' perception of children's mental capacities would increase as a function of the target child's age, with participants generally attributing more and greater abilities to older children.

(2) We predicted that, in the domains of *cognition and control* and *social connection*, participants would perceive relatively more dramatic developmental changes extending later into childhood (i.e., lower intercepts and steeper slopes), whereas in the domains of *bodily sensation* and *negative affect* participants would perceive relatively smaller changes across development (i.e., higher intercepts and shallower slopes).

To test these hypotheses, we conducted a multilevel logistic regression via a generalized additive model, regressing item-level responses onto the domain (i.e., which of the four "factors" this capacity was selected to represented) and a spline-based smooth of the target age of the child fitted separately for each domain, including the maximal random effects structure that would allow the model to converge. Using the "mgcv" package for R (Wood, 2017), we specified this model as follows: *gam(response ~ s(target age in years, by = domain) + domain, random = list(subject ID = ~1, capacity = ~1).* Attempting to model random slopes for domain or for target age resulted in pathological models. Domain was dummy-coded with *cognition and control* as the baseline. The full results of this model are presented in Supplementary Tables 13 and 14; see Figure 2, Panel A (main text) for a visualization of these perceived trajectories.

In brief, we view these results as confirming our hypotheses that US adults would rate older children as more capable across the board, but that perceptions of the development of children's capacities would differ across domains. (See main text for extended interpretation.)

Table 13: Study 2, GAM results: Parametric coefficients (fixed effects).

Parameter	Coefficient (log-odds)	95% CI	SE	z	p
Cognition and control (intercept)	-1.19	[-1.53, -0.86]	0.17	-7.00	<0.001
Bodily sensation vs. Cognition and control	4.15	[3.71, 4.59]	0.22	18.46	<0.001
Negative affect vs. Cognition and control	2.55	[2.11, 2.98]	0.22	11.39	<0.001
Social connection vs. Cognition and control	2.79	[2.35, 3.23]	0.22	12.47	<0.001

Table 14: Study 2, GAM results: Approximate significance of smooth terms.

Parameter	z	Estimated df	p
Target year: Bodily sensation	281.51	3.86	<0.001
Target year: Negative affect	1568.47	5.03	<0.001
Target year: Social connection	3196.67	6.93	<0.001
Target year: Cognition and control	4537.42	5.79	<0.001
Capacity (random effect)	861.08	15.69	<0.001
Participant (random effect)	10959.17	294.74	<0.001

Study 3

As described in the main text, Study 3 was designed with two goals in mind: First, we aimed to provide a more direct assessment of the differences in perceived developmental trajectories surfaced by Study 2. Second, we aimed to probe the intuitive theories that might underlie the differentiation of the four factors surfaced by Studies 1-2 and the perceived developmental trajectories surfaced by Study 2 (and the current study).

In the main text, we presented the results of a generalized additive model (GAM) predicting participants' capacity ratings as a function of target age, domain, and interactions between them; and briefly presented the results of a series of multilevel linear regression models predicting participants' ratings of developmental mechanisms as a function of domain; and provided visualizations of participants' selections of the "most important" mechanism for each capacity, by domain. Here, we present the full results of all of these models, as well as the results of a multilevel logistic regression model of the selection of "most important" mechanism.

Capacity ratings: Developmental trajectories

We preregistered two hypotheses about the perceived developmental trajectories we anticipated to observe in Study 3 (identical to our hypotheses for Study 2):

(1) We predicted that, on average, participants' perception of children's mental capacities would increase as a function of the target child's age, with participants generally attributing more and greater abilities to older children.

(2) We predicted that, in the domains of *cognition and control* and *social connection*, participants would perceive relatively more dramatic developmental changes extending later into childhood (i.e., lower intercepts and steeper slopes), whereas in the domains of *bodily sensation* and *negative affect* participants would perceive relatively smaller changes across development (i.e., higher intercepts and shallower slopes).

Following Study 2, to test these hypotheses we conducted a multilevel logistic regression via a generalized additive model, regressing item-level responses onto the domain and a spline-based smooth of the target age of the child fitted separately for each domain, including the maximal random effects structure that would allow the model to converge. Using the "mgcv" package for R (Wood, 2017), we specified this

model as follows: *gam(responses ~ s(target age in years, by = domain) + domain + s(subject ID, bs = "re") + s(capacity, bs = "re")*. Again, attempting to model random slopes for domain or for target age resulted in pathological models. Again, domain was dummy-coded with *cognition and control* as the baseline. The full results of this model are presented in Supplementary Tables 15 and 16; see Figure 2, Panel B (main text) for a visualization of these perceived trajectories.

In brief, we view these results as confirming our hypotheses that US adults would rate older children as more capable across the board, but that perceptions of the development of children's capacities would differ across domains. (See main text for extended interpretation.)

Table 15: Study 3, GAM results: Parametric coefficients (fixed effects).

Parameter	Coefficient (log-odds)	95% CI	SE	z	p
Cognition and control (intercept)	-1.26	[-2.34, -0.18]	0.55	-2.29	0.022
Bodily sensation vs. Cognition and control	4.82	[3.29, 6.34]	0.78	6.20	<0.001
Negative affect vs. Cognition and control	2.53	[1.02, 4.05]	0.78	3.27	0.001
Social connection vs. Cognition and control	2.53	[1.02, 4.05]	0.78	3.27	0.001

Table 16: Study 3, GAM results: Approximate significance of smooth terms.

Parameter	z	Estimated df	p
Target year: Bodily sensation	110.98	3.85	<0.001
Target year: Negative affect	480.78	4.24	<0.001
Target year: Social connection	1641.65	6.89	<0.001
Target year: Cognition and control	1597.68	5.71	<0.001
Capacity (random effect)	1315.04	3.98	<0.001
Participant (random effect)	5120.13	274.61	<0.001

Figure 2

Developmental mechanisms: Ratings

Summary tables

Regression models To supplement our visual assessment of which developmental mechanisms were considered to be the most or least important in the development of each of the individual mental capacities included in this study (*bodily sensation*, *negative affect*, *social connection*, and *cognition and control*), we conducted a series of multilevel linear regression models, regressing responses to this question onto domain, including the maximal random effects structure that allowed the model to converge. Using the "lme4" package for R (Bates et al., 2015), we specified these models as follows: *lmer(response ~ domain + (1 | participant) + (1 | capacity)*. In these analyses, domain and developmental mechanism were both effect-coded for comparisons to the grand mean collapsing across domains and mechanisms.

The full results of these models are presented in Supplementary Tables 17, 18, 19, 20, and 21; see also Figure 3 (main text).

All domains In brief, this analysis suggested that the perceived importance of these developmental mechanisms varied both overall (see main effects) and across domains (see interaction terms).

Table 17: Study 3, multilevel linear model results (all domains): Fixed effects.

Parameter	Coefficient	95% CI	SE	t	df	p
Grand mean (GM) (intercept)	0.00	[-0.10, 0.10]	0.05	0.00	24037	1.000
Mechanism (Biologically 'preprogrammed' vs. GM)	0.30	[0.27, 0.33]	0.01	20.90	24037	<0.001
Mechanism (Experiences in the womb vs. GM)	-0.69	[-0.72, -0.67]	0.01	-48.35	24037	<0.001
Mechanism (Body grows and matures vs. GM)	-0.29	[-0.32, -0.26]	0.01	-20.42	24037	<0.001
Mechanism (Brain changes vs. GM)	0.40	[0.37, 0.43]	0.01	27.93	24037	<0.001
Mechanism (Observes objects and physical world vs. GM)	0.04	[0.01, 0.06]	0.01	2.45	24037	0.014
Mechanism (Observes people vs. GM)	0.24	[0.21, 0.27]	0.01	16.91	24037	<0.001
Mechanism (Interacts with people vs. GM)	0.27	[0.25, 0.30]	0.01	19.11	24037	<0.001
Mechanism (People explicitly teach vs. GM)	-0.16	[-0.19, -0.13]	0.01	-10.97	24037	<0.001
Mechanism (Actively experiments vs. GM)	-0.05	[-0.08, -0.02]	0.01	-3.33	24037	<0.001
Bodily sensation (vs. GM)	-0.34	[-0.51, -0.18]	0.08	-4.11	24037	<0.001
Social connection (vs. GM)	0.28	[0.12, 0.45]	0.08	3.40	24037	<0.001
Cognition and control (vs. GM)	0.17	[0.01, 0.34]	0.08	2.07	24037	0.038
Mechanism (Biologically 'preprogrammed' vs. GM) x Bodily sensation (vs. GM)	1.06	[1.01, 1.11]	0.02	42.83	24037	<0.001
Mechanism (Experiences in the womb vs. GM) x Bodily sensation (vs. GM)	0.65	[0.60, 0.69]	0.02	26.01	24037	<0.001
Mechanism (Body grows and matures vs. GM) x Bodily sensation (vs. GM)	0.49	[0.44, 0.54]	0.02	19.72	24037	<0.001
Mechanism (Brain changes vs. GM) x Bodily sensation (vs. GM)	-0.19	[-0.24, -0.14]	0.02	-7.71	24037	<0.001
Mechanism (Observes objects and physical world vs. GM) x Bodily sensation (vs. GM)	-0.37	[-0.42, -0.32]	0.02	-14.92	24037	<0.001
Mechanism (Observes people vs. GM) x Bodily sensation (vs. GM)	-0.52	[-0.57, -0.47]	0.02	-20.90	24037	<0.001
Mechanism (Interacts with people vs. GM) x Bodily sensation (vs. GM)	-0.57	[-0.62, -0.52]	0.02	-23.10	24037	<0.001
Mechanism (People explicitly teach vs. GM) x Bodily sensation (vs. GM)	-0.35	[-0.40, -0.30]	0.02	-14.18	24037	<0.001
Mechanism (Actively experiments vs. GM) x Bodily sensation (vs. GM)	-0.31	[-0.36, -0.26]	0.02	-12.61	24037	<0.001
Mechanism (Biologically 'preprogrammed' vs. GM) x Social connection (vs. GM)	-0.24	[-0.29, -0.19]	0.02	-9.72	24037	<0.001
Mechanism (Experiences in the womb vs. GM) x Social connection (vs. GM)	-0.28	[-0.33, -0.23]	0.02	-11.35	24037	<0.001
Mechanism (Body grows and matures vs. GM) x Social connection (vs. GM)	-0.18	[-0.23, -0.14]	0.02	-7.42	24037	<0.001
Mechanism (Brain changes vs. GM) x Social connection (vs. GM)	0.01	[-0.03, 0.06]	0.02	0.60	24037	0.552
Mechanism (Observes objects and physical world vs. GM) x Social connection (vs. GM)	0.12	[0.07, 0.17]	0.02	4.75	24037	<0.001
Mechanism (Observes people vs. GM) x Social connection (vs. GM)	0.18	[0.13, 0.23]	0.02	7.25	24037	<0.001
Mechanism (Interacts with people vs. GM) x Social connection (vs. GM)	0.24	[0.19, 0.29]	0.02	9.62	24037	<0.001
Mechanism (People explicitly teach vs. GM) x Social connection (vs. GM)	0.05	[0.00, 0.10]	0.02	1.98	24037	0.048
Mechanism (Actively experiments vs. GM) x Social connection (vs. GM)	0.13	[0.08, 0.17]	0.02	5.08	24037	<0.001
Mechanism (Biologically 'preprogrammed' vs. GM) x Cognition and control (vs. GM)	-0.93	[-0.98, -0.88]	0.02	-37.58	24037	<0.001
Mechanism (Experiences in the womb vs. GM) x Cognition and control (vs. GM)	-0.50	[-0.55, -0.45]	0.02	-20.12	24037	<0.001
Mechanism (Body grows and matures vs. GM) x Cognition and control (vs. GM)	-0.26	[-0.31, -0.21]	0.02	-10.57	24037	<0.001
Mechanism (Brain changes vs. GM) x Cognition and control (vs. GM)	0.18	[0.13, 0.23]	0.02	7.13	24037	<0.001
Mechanism (Observes objects and physical world vs. GM) x Cognition and control (vs. GM)	0.11	[0.06, 0.16]	0.02	4.31	24037	<0.001
Mechanism (Observes people vs. GM) x Cognition and control (vs. GM)	0.26	[0.21, 0.31]	0.02	10.51	24037	<0.001
Mechanism (Interacts with people vs. GM) x Cognition and control (vs. GM)	0.27	[0.22, 0.32]	0.02	10.92	24037	<0.001
Mechanism (People explicitly teach vs. GM) x Cognition and control (vs. GM)	0.60	[0.55, 0.65]	0.02	24.23	24037	<0.001
Mechanism (Actively experiments vs. GM) x Cognition and control (vs. GM)	0.42	[0.38, 0.47]	0.02	17.08	24037	<0.001

Bodily sensation For capacities in the domain of *bodily sensation*, this analysis suggested that participants rated biological preprogramming, body changes, and brain changes as more important drivers of development than other mechanisms; see also Figure 3 (main text).

Table 18: Study 3, multilevel linear model results (bodily sensation): Fixed effects.

Parameter	Coefficient	95% CI	SE	t	df	p
Grand mean (GM) (intercept)	0.00	[-0.07, 0.07]	0.03	0.00	6007	1.000
Mechanism (Biologically 'preprogrammed' vs. GM)	1.31	[1.26, 1.36]	0.03	51.34	6007	<0.001
Mechanism (Experiences in the womb vs. GM)	-0.05	[-0.10, 0.00]	0.03	-1.78	6007	0.075
Mechanism (Body grows and matures vs. GM)	0.19	[0.14, 0.24]	0.03	7.42	6007	<0.001
Mechanism (Brain changes vs. GM)	0.20	[0.15, 0.25]	0.03	7.86	6007	<0.001
Mechanism (Observes objects and physical world vs. GM)	-0.32	[-0.37, -0.27]	0.03	-12.63	6007	<0.001
Mechanism (Observes people vs. GM)	-0.27	[-0.32, -0.22]	0.03	-10.41	6007	<0.001
Mechanism (Interacts with people vs. GM)	-0.29	[-0.34, -0.24]	0.03	-11.28	6007	<0.001
Mechanism (People explicitly teach vs. GM)	-0.49	[-0.54, -0.44]	0.03	-19.19	6007	<0.001
Mechanism (Actively experiments vs. GM)	-0.35	[-0.40, -0.30]	0.03	-13.59	6007	<0.001

Negative affect For capacities in the domain of *negative affect*, this analysis suggested that participants rated biological preprogramming, brain changes, social interactions, and observations of other people as more important drivers of development than other mechanisms; see also Figure 3 (main text).

Table 19: Study 3, multilevel linear model results (negative affect): Fixed effects.

Parameter	Coefficient	95% CI	SE	t	df	p
Grand mean (GM) (intercept)	0.00	[-0.06, 0.06]	0.03	0.00	6007	1.000
Mechanism (Biologically 'preprogrammed' vs. GM)	0.42	[0.36, 0.48]	0.03	13.87	6007	<0.001
Mechanism (Experiences in the womb vs. GM)	-0.58	[-0.64, -0.52]	0.03	-18.83	6007	<0.001
Mechanism (Body grows and matures vs. GM)	-0.35	[-0.41, -0.29]	0.03	-11.34	6007	<0.001
Mechanism (Brain changes vs. GM)	0.41	[0.35, 0.47]	0.03	13.52	6007	<0.001
Mechanism (Observes objects and physical world vs. GM)	0.19	[0.13, 0.25]	0.03	6.10	6007	<0.001
Mechanism (Observes people vs. GM)	0.33	[0.27, 0.39]	0.03	10.82	6007	<0.001
Mechanism (Interacts with people vs. GM)	0.35	[0.29, 0.41]	0.03	11.40	6007	<0.001
Mechanism (People explicitly teach vs. GM)	-0.47	[-0.53, -0.41]	0.03	-15.40	6007	<0.001
Mechanism (Actively experiments vs. GM)	-0.29	[-0.35, -0.23]	0.03	-9.63	6007	<0.001

Social connection For capacities in the domain of *social connection*, this analysis suggested that participants rated social interactions, observations of other people, and brain changes as more important drivers of development than other mechanisms; see also Figure 3 (main text).

Table 20: Study 3, multilevel linear model results (social connection): Fixed effects.

Parameter	Coefficient	95% CI	SE	t	df	p
Grand mean (GM) (intercept)	0.00	[-0.37, 0.37]	0.19	0.00	6007	1.000
Mechanism (Biologically 'preprogrammed' vs. GM)	0.06	[0.01, 0.12]	0.03	2.14	6007	0.032
Mechanism (Experiences in the womb vs. GM)	-1.05	[-1.11, -0.99]	0.03	-35.82	6007	<0.001
Mechanism (Body grows and matures vs. GM)	-0.51	[-0.57, -0.46]	0.03	-17.53	6007	<0.001
Mechanism (Brain changes vs. GM)	0.45	[0.39, 0.51]	0.03	15.25	6007	<0.001
Mechanism (Observes objects and physical world vs. GM)	0.17	[0.11, 0.22]	0.03	5.63	6007	<0.001
Mechanism (Observes people vs. GM)	0.46	[0.40, 0.51]	0.03	15.53	6007	<0.001
Mechanism (Interacts with people vs. GM)	0.55	[0.50, 0.61]	0.03	18.85	6007	<0.001
Mechanism (People explicitly teach vs. GM)	-0.12	[-0.17, -0.06]	0.03	-3.97	6007	<0.001
Mechanism (Actively experiments vs. GM)	0.08	[0.03, 0.14]	0.03	2.88	6007	0.004

Cognition and control For capacities in the domain of *cognition and control*, this analysis suggested that participants rated brain changes, social interactions, observations of other people, explicit teaching, and active experimentation as more important drivers of development than other mechanisms; see also Figure 3 (main text).

Table 21: Study 3, multilevel linear model results (cognition and control): Fixed effects.

Parameter	Coefficient	95% CI	SE	t	df	p
Grand mean (GM) (intercept)	0.00	[-0.17, 0.17]	0.09	0.00	6007	1.000
Mechanism (Biologically 'preprogrammed' vs. GM)	-0.67	[-0.73, -0.62]	0.03	-24.82	6007	<0.001
Mechanism (Experiences in the womb vs. GM)	-1.27	[-1.32, -1.22]	0.03	-46.73	6007	<0.001
Mechanism (Body grows and matures vs. GM)	-0.59	[-0.64, -0.54]	0.03	-21.75	6007	<0.001
Mechanism (Brain changes vs. GM)	0.62	[0.56, 0.67]	0.03	22.62	6007	<0.001
Mechanism (Observes objects and physical world vs. GM)	0.15	[0.10, 0.20]	0.03	5.57	6007	<0.001
Mechanism (Observes people vs. GM)	0.54	[0.48, 0.59]	0.03	19.72	6007	<0.001
Mechanism (Interacts with people vs. GM)	0.58	[0.53, 0.63]	0.03	21.36	6007	<0.001
Mechanism (People explicitly teach vs. GM)	0.47	[0.42, 0.53]	0.03	17.41	6007	<0.001
Mechanism (Actively experiments vs. GM)	0.40	[0.35, 0.45]	0.03	14.75	6007	<0.001

Developmental mechanisms: Selection of "most important"

As described in the main text, we also assessed participants' responses to forced-choice questions about the "most important" factor driving the development of each of these capacities.

Summary tables

Multinomial regression Here we present a multinomial log-linear model to predict the likelihood of selecting a mechanism by capacity Using the "nnet" package for R (Venables & Ripley, 2002), we specified this model as follows: *multinom(choice of extrinsic mechanism ~ capacity.* (Note that this model was not equipped to represent the within-subjects nature of this dataset, and that capacities are not nested within domain for these purposes.) In this analysis, capacity was effect-coded for comparisons to the grand mean collapsing across capacities. The full results of this model are presented in Supplementary Table 22.

This analysis aligned with our observation that participants' choice of mechanisms as the "most important" driver of development varied by capacity.

Table 22: Study 3, multinomial log linear model results.

Response	Parameter	Coefficient	95% CI	SE	z	p
biologically 'preprogrammed'	Grand mean (GM) (intercept)	5.16	[4.52, 5.80]	0.33	15.85	<0.001
	Capacity (Feeling pain vs. GM)	0.35	[-1.43, 2.13]	0.91	0.39	0.700
	Capacity (Getting hungry vs. GM)	0.39	[-1.40, 2.18]	0.91	0.43	0.670
	Capacity (Feeling distressed vs. GM)	-1.06	[-2.21, 0.10]	0.59	-1.79	0.073
	Capacity (Feeling happy vs. GM)	-1.18	[-2.53, 0.16]	0.69	-1.73	0.084
	Capacity (Learning from other people vs. GM)	-1.50	[-3.29, 0.29]	0.91	-1.64	0.101
	Capacity (Controlling their emotions vs. GM)	-2.52	[-4.35, -0.69]	0.93	-2.70	0.007
	Capacity (Reasoning about things vs. GM)	7.46	[7.03, 7.88]	0.22	34.49	<0.001
experiences in the womb	Grand mean (GM) (intercept)	2.63	[1.94, 3.32]	0.35	7.44	<0.001
	Capacity (Feeling pain vs. GM)	-0.69	[-2.59, 1.22]	0.97	-0.71	0.479
	Capacity (Getting hungry vs. GM)	-0.43	[-2.32, 1.46]	0.96	-0.45	0.656
	Capacity (Feeling distressed vs. GM)	-1.94	[-3.30, -0.57]	0.70	-2.78	0.005
	Capacity (Feeling happy vs. GM)	-1.13	[-2.60, 0.35]	0.75	-1.50	0.134
	Capacity (Learning from other people vs. GM)	-1.02	[-2.97, 0.92]	0.99	-1.03	0.304
	Capacity (Controlling their emotions vs. GM)	-0.84	[-2.76, 1.09]	0.98	-0.85	0.394
	Capacity (Reasoning about things vs. GM)	8.38	[7.54, 9.22]	0.43	19.62	<0.001
body grows and matures	Grand mean (GM) (intercept)	2.86	[2.18, 3.55]	0.35	8.20	<0.001
	Capacity (Feeling pain vs. GM)	-0.92	[-2.83, 0.98]	0.97	-0.95	0.342
	Capacity (Getting hungry vs. GM)	0.08	[-1.76, 1.93]	0.94	0.09	0.930
	Capacity (Feeling distressed vs. GM)	-1.88	[-3.20, -0.57]	0.67	-2.80	0.005
	Capacity (Feeling happy vs. GM)	-1.48	[-2.96, 0.00]	0.76	-1.96	0.051
	Capacity (Learning from other people vs. GM)	-1.77	[-3.81, 0.27]	1.04	-1.70	0.089
	Capacity (Controlling their emotions vs. GM)	-1.07	[-2.99, 0.85]	0.98	-1.09	0.274
	Capacity (Reasoning about things vs. GM)	8.70	[8.04, 9.37]	0.34	25.65	<0.001
	Grand mean (GM) (intercept)	4.49	[3.83, 5.14]	0.33	13.42	<0.001
	Capacity (Feeling pain vs. GM)	-2.09	[-3.94, -0.24]	0.95	-2.21	0.027
	Capacity (Getting hungry vs. GM)	-3.38	[-5.42, -1.34]	1.04	-3.25	0.001
	Capacity (Feeling distressed vs. GM)	-1.97	[-3.16, -0.78]	0.61	-3.25	0.001
	Capacity (Feeling happy vs. GM)	-1.02	[-2.38, 0.34]	0.69	-1.47	0.141

Table 22: Study 3, multinomial log linear model results. *(continued)*

Response	Parameter	Coefficient	95% CI	SE	z	p
brain changes	Capacity (Learning from other people vs. GM)	-0.24	[-2.03, 1.55]	0.91	-0.26	0.794
	Capacity (Controlling their emotions vs. GM)	-0.03	[-1.82, 1.76]	0.91	-0.03	0.974
	Capacity (Reasoning about things vs. GM)	9.97	[9.66, 10.29]	0.16	62.27	<0.001
senses improve	Grand mean (GM) (intercept)	3.09	[2.42, 3.76]	0.34	9.01	<0.001
	Capacity (Feeling pain vs. GM)	-0.46	[-2.30, 1.39]	0.94	-0.49	0.627
	Capacity (Getting hungry vs. GM)	-1.48	[-3.43, 0.47]	1.00	-1.49	0.137
	Capacity (Feeling distressed vs. GM)	-1.63	[-2.88, -0.37]	0.64	-2.53	0.011
	Capacity (Feeling happy vs. GM)	-1.30	[-2.73, 0.14]	0.73	-1.77	0.076
	Capacity (Learning from other people vs. GM)	-0.79	[-2.65, 1.07]	0.95	-0.83	0.406
	Capacity (Controlling their emotions vs. GM)	-0.79	[-2.65, 1.08]	0.95	-0.83	0.408
	Capacity (Reasoning about things vs. GM)	7.92	[7.10, 8.74]	0.42	18.86	<0.001
observes objects and physical world	Grand mean (GM) (intercept)	3.13	[2.44, 3.83]	0.36	8.82	<0.001
	Capacity (Feeling pain vs. GM)	-2.44	[-4.60, -0.29]	1.10	-2.22	0.026
	Capacity (Getting hungry vs. GM)	-2.44	[-4.60, -0.27]	1.11	-2.20	0.028
	Capacity (Feeling distressed vs. GM)	-1.24	[-2.48, 0.00]	0.63	-1.95	0.051
	Capacity (Feeling happy vs. GM)	-1.43	[-2.88, 0.03]	0.74	-1.92	0.054
	Capacity (Learning from other people vs. GM)	-0.30	[-2.14, 1.54]	0.94	-0.32	0.749
	Capacity (Controlling their emotions vs. GM)	-1.34	[-3.26, 0.58]	0.98	-1.36	0.172
	Capacity (Reasoning about things vs. GM)	9.89	[9.44, 10.34]	0.23	43.23	<0.001
observes people	Grand mean (GM) (intercept)	3.66	[2.98, 4.33]	0.34	10.61	<0.001
	Capacity (Feeling pain vs. GM)	-2.28	[-4.26, -0.30]	1.01	-2.26	0.024
	Capacity (Getting hungry vs. GM)	-2.96	[-5.12, -0.80]	1.10	-2.69	0.007
	Capacity (Feeling distressed vs. GM)	-2.19	[-3.45, -0.93]	0.64	-3.41	<0.001
	Capacity (Feeling happy vs. GM)	-0.89	[-2.27, 0.50]	0.71	-1.25	0.210
	Capacity (Learning from other people vs. GM)	0.50	[-1.30, 2.30]	0.92	0.54	0.587
	Capacity (Controlling their emotions vs. GM)	-0.05	[-1.86, 1.76]	0.92	-0.05	0.959
	Capacity (Reasoning about things vs. GM)	9.10	[8.65, 9.55]	0.23	39.62	<0.001
	Grand mean (GM) (intercept)	1.76	[1.08, 2.43]	0.35	5.08	<0.001
	Capacity (Feeling pain vs. GM)	-0.66	[-2.65, 1.33]	1.01	-0.65	0.514

Table 22: Study 3, multinomial log linear model results. *(continued)*

Response	Parameter	Coefficient	95% CI	SE	z	p
interacts with people	Capacity (Getting hungry vs. GM)	-16.32	[-16.47, -16.17]	0.08	-216.53	<0.001
	Capacity (Feeling distressed vs. GM)	-0.29	[-1.53, 0.95]	0.63	-0.46	0.645
	Capacity (Feeling happy vs. GM)	1.42	[0.08, 2.77]	0.69	2.07	0.038
	Capacity (Learning from other people vs. GM)	2.37	[0.62, 4.12]	0.90	2.65	0.008
	Capacity (Controlling their emotions vs. GM)	1.88	[0.12, 3.65]	0.90	2.09	0.036
	Capacity (Reasoning about things vs. GM)	10.96	[10.48, 11.44]	0.24	44.92	<0.001
people explicitly teach	Grand mean (GM) (intercept)	-0.95	[-1.73, -0.17]	0.40	-2.38	0.017
	Capacity (Feeling pain vs. GM)	-12.34	[-12.52, -12.16]	0.09	-135.15	<0.001
	Capacity (Getting hungry vs. GM)	-12.87	[-13.04, -12.69]	0.09	-140.93	<0.001
	Capacity (Feeling distressed vs. GM)	0.54	[-1.09, 2.18]	0.83	0.65	0.513
	Capacity (Feeling happy vs. GM)	0.26	[-1.82, 2.34]	1.06	0.24	0.808
	Capacity (Learning from other people vs. GM)	3.95	[2.18, 5.71]	0.90	4.39	<0.001
	Capacity (Controlling their emotions vs. GM)	5.20	[3.46, 6.94]	0.89	5.86	<0.001
	Capacity (Reasoning about things vs. GM)	13.79	[13.17, 14.41]	0.32	43.73	<0.001
actively experiments	Grand mean (GM) (intercept)	2.98	[2.29, 3.67]	0.35	8.45	<0.001
	Capacity (Feeling pain vs. GM)	-1.89	[-3.93, 0.16]	1.04	-1.81	0.070
	Capacity (Getting hungry vs. GM)	-1.88	[-3.93, 0.17]	1.05	-1.80	0.073
	Capacity (Feeling distressed vs. GM)	-2.69	[-4.15, -1.24]	0.74	-3.64	<0.001
	Capacity (Feeling happy vs. GM)	-1.73	[-3.23, -0.23]	0.77	-2.25	0.024
	Capacity (Learning from other people vs. GM)	-0.68	[-2.55, 1.19]	0.95	-0.71	0.476
	Capacity (Controlling their emotions vs. GM)	0.32	[-1.51, 2.14]	0.93	0.34	0.734
	Capacity (Reasoning about things vs. GM)	10.33	[9.91, 10.75]	0.22	48.04	<0.001

In brief, this analysis suggested that selections of what we would call "social" mechanisms of development (explicit teaching and interactions with people) were particularly uncommon for capacities in the domain of *bodily sensations*, and particularly common for capacities in the domain of *cognition and control* (as well as one of the capacities in the domain of *social connection*). More broadly, many of the developmental mechanisms were selected more often for capacities in the domain of *cognition and control* than capacities in other domains, reflecting the greater diversity of choices in this domain, including the (occasional) selection of mechanisms that were rarely if ever selected as 'most important' in other domains (e.g., active experimentation on the part of the child).

Study comparison

As described in the main text, all of the current studied employed a fixed-order design, in which all participants assessed target ages in a fixed order beginning with the youngest target age (newborns) and proceeding chronologically until the oldest target age (5-year-olds). This decision reflects the fact that our priority in these studies was to assess beliefs about the developmental trajectories of capacities, rather than beliefs about capacities at a specific point in development. However, one limitation of this fixed-order design is that it makes it difficult to assess the stability of participants' assessments of the fine-grained differences between specific points in development.

To provide some sense of the stability and reliability of US adults' representations of specific target ages across studies, Supplementary Figure 9 presents a comparison of capacity ratings at the three target ages that were included in all of the current studies (newborns, 9-month-olds, and 5-year-olds). As a reminder, in Study 1, participants assessed 60 capacities (including but not limited to the 20 capacities depicted in Panel A), at each of the 3 target ages depicted here. In Study 2, participants assessed the 20 capacities depicted in Panel A, at 13 target ages (including but not limited to the 3 target ages depicted here). In Study 3, participants assessed 8 capacities (a subset of the 20 capacities depicted in Panel A), at 13 target ages (including but not limited to the 3 target ages depicted here).

Selection of capacities: Comparison to previous studies

Supplementary Table 23 provides an item-by-item comparison of the capacities used here to those used in previous studies of mind perception (Gray et al., 2007; Malle, 2019; Weisman et al., 2017b, 2017a).

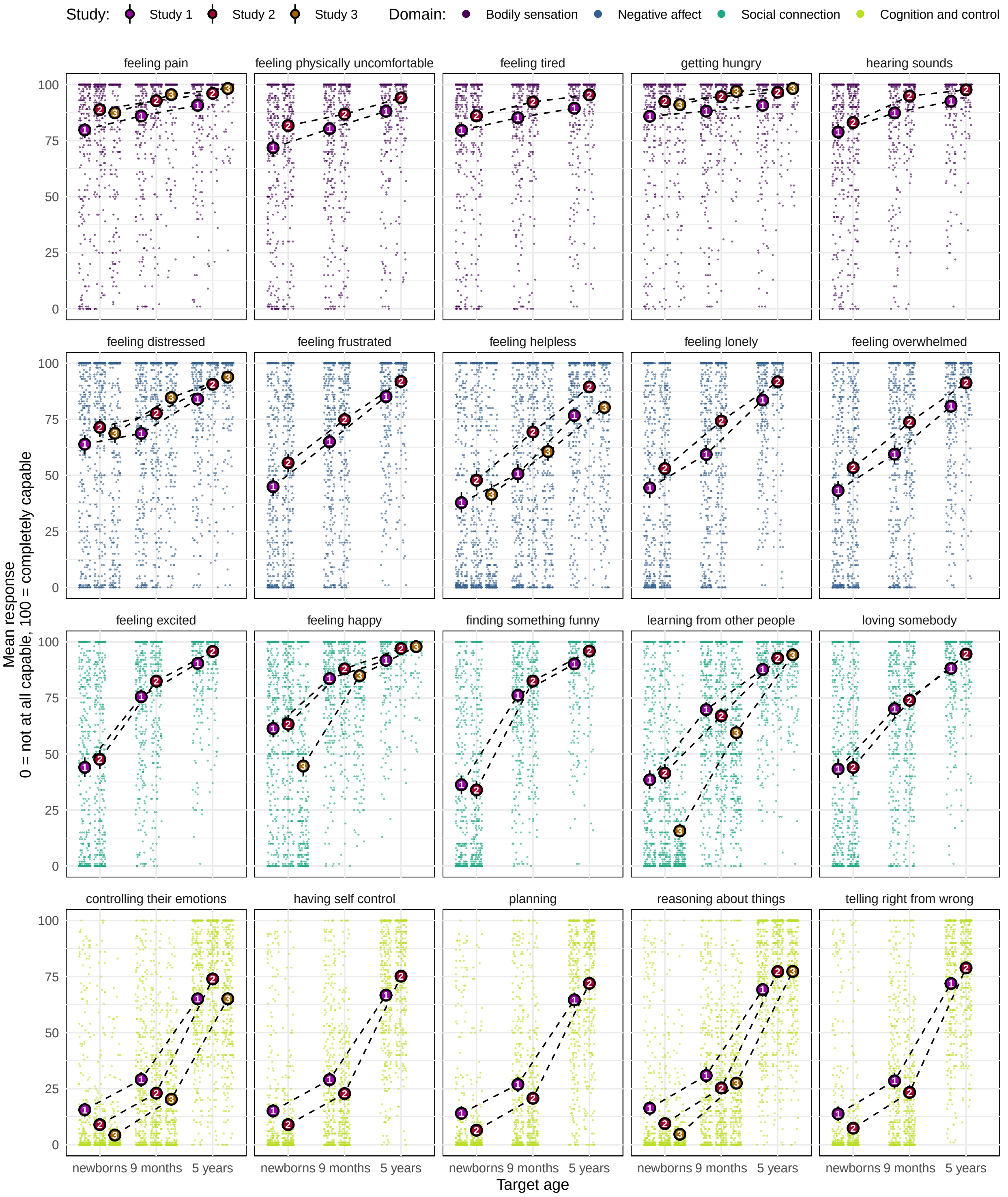

Figure S9: Comparison of capacity ratings across studies (Studies 1-3). This visualization includes responses for each of the capacities and target ages held in common across at least two of our three studies. Lighter colorful points represent individual participants' responses, larger points correspond to mean responses across the sample, and error bars are bootstrapped 95% confidence intervals.

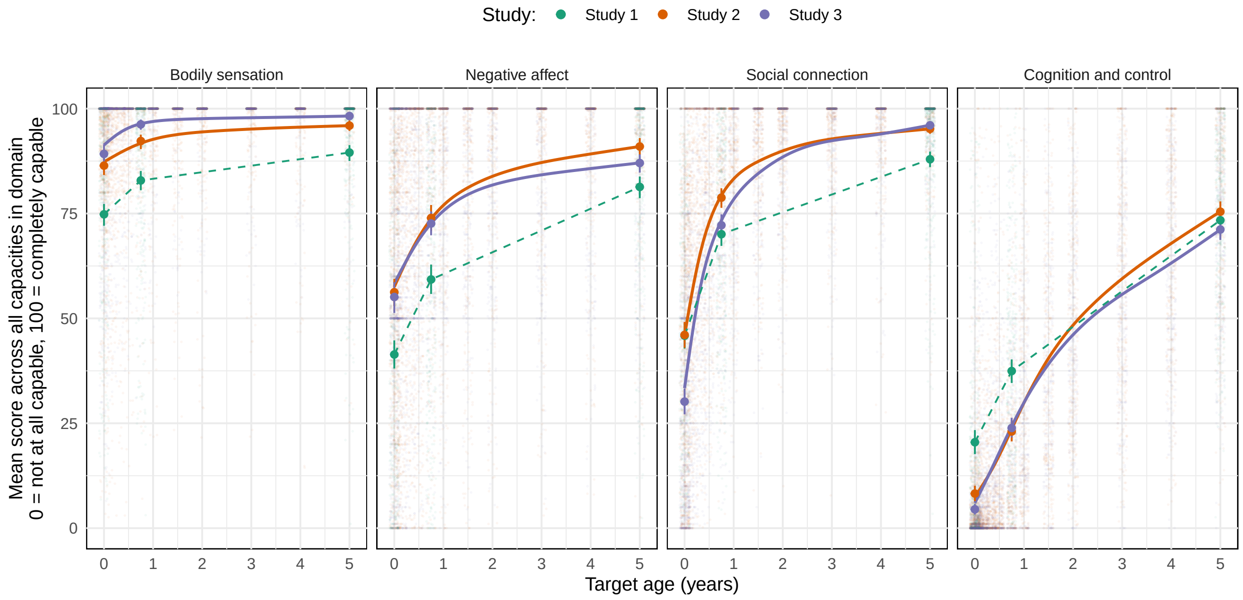

Figure S10: Comparison of mean scores across studies (Studies 1-3). These scores incorporate responses to all of the capacities and target ages included in each study; this varies across studies. Lighter points represent individual participants' scores, larger points correspond to mean scores across the sample, and error bars are bootstrapped 95% confidence intervals. The dotted line for Study 1 connects mean scores across target ages; the solid lines for Studies 2-3 reflect predictions from study-specific GAMs.

Table 23: Item-by-item comparison of the capacities included in the current studies to capacities included in previous studies of mind perception.

Current studies	Previous studies				
Study 1	Weisman et al. (2017, PNAS)	Weisman et al. (2017, Proc. CogSci)	Gray et al. (2007)	Malle (2019, Study 1)	Malle (2019, Study 2)
**Bodily sensation**					
getting hungry[*†]	getting hungry	get hungry	hunger	can feel hunger	being hungry
feeling pain[*†]	experiencing pain	feel pain	pain	can be in physical pain	feeling pain
feeling tired[*]	feeling tired	feel tired	-	has a need for sleep	-
feeling thirsty	-	-	-	-	can feel thirsty
feeling too hot or too cold	-	-	-	-	-
feeling physically uncomfortable[*]	-	-	-	-	-
hearing sounds[*]	detecting sounds	hear sounds	-	[similar to 'can see or hear things']	[similar to 'seeing and hearing the world around them']
being comforted by physical touch	-	-	-	-	-
feeling distressed[*†]	-	-	-	-	-
seeing	seeing things	see things	-	[similar to 'can see or hear things']	[similar to 'seeing and hearing the world around them']
feeling calm	feeling calm	feel calm	-	-	-
**Negative affect**					
feeling helpless[*†]	-	-	-	-	-
feeling overwhelmed[*]	-	-	-	-	-
feeling frustrated[*]	-	-	-	-	-
feeling annoyed	-	-	-	-	-
feeling neglected	-	-	-	-	-
**Social connection**					
feeling excited[*]	-	-	-	-	-
finding something funny[*]	-	-	-	-	-
loving somebody[*]	-	-	-	-	loving specific people
learning from other people[*†]	-	-	-	-	[similar to 'learning by imitation']
feeling happy[*†]	feeling happy	feel happy	[similar to 'joy']	-	feeling happy
feeling loved	feeling love	feel love	-	-	-
recognizing somebody else	recognizing someone	recognize somebody else	-	-	-
getting pleasure from music	-	-	-	-	-
being afraid of somebody	-	-	-	-	[similar to 'disliking people']
listening to somebody	-	-	-	-	-

Table 23: Item-by-item comparison of the capacities included in the current studies to capacities included in previous studies of mind perception. *(continued)*

Study 1	Weisman et al. (2017, PNAS)	Weisman et al. (2017, Proc. CogSci)	Gray et al. (2007)	Malle (2019, Study 1)	Malle (2019, Study 2)
having thoughts	having thoughts	have thoughts	thought	-	-
feeling sad	[similar to ‘feeling depressed’]	feel sad	-	-	-
feeling safe	feeling safe	feel safe	-	-	-
feeling textures (for example, smooth, rough)	-	-	-	-	-
getting angry	getting angry	get angry	rage	can be angry	getting angry
feeling pleasure	experiencing pleasure	feel pleasure	pleasure	can experience pleasure	feeling pleasure
being angry at somebody	-	-	-	-	[similar to ‘disliking people’]
feeling lonely[*]	-	-	-	-	-
feeling bored	-	-	-	-	-
feeling confused	-	-	-	-	-
feeling scared	experiencing fear	feel scared	fear	-	-
being aware of things	[similar to ‘being conscious’]	be aware of things	[similar to ‘consciousness’]	[similar to ‘can perceive things’]	-
**Cognition and control**					
planning[*]	[similar to ‘working toward a goal’]	[similar to ‘have goals’]	planning	can plan for the future	planning for the future
having self-control[*]	[similar to ‘exercising self-restraint’]	have self-control	self-control	can exercise self-control	excersing self-control
thinking before they act	-	-	-	can deliberate	deliberating before acting
having goals	working toward a goal	have goals	[similar to ‘planning’]	-	setting goals
reasoning about things[*†]	reasoning about things	[similar to ‘figure out how to do things’]	-	can reason logically	reasoning logically
controlling their emotions[*†]	-	-	-	-	-
telling right from wrong[*]	telling right from wrong	[similar to ‘know what’s nice and what’s mean’]	morality	[similar to ‘may deserve praise or blame’ and ’may deserve punishment’]	[similar to ‘upholding moral values’]
understanding what somebody else is thinking	-	-	-	-	understanding others’ minds
focusing on a goal	-	-	-	-	-
feeling guilty	experiencing guilt	feel guilty	-	[similar to ‘can feel shame or pride’]	-

Table 23: Item-by-item comparison of the capacities included in the current studies to capacities included in previous studies of mind perception. *(continued)*

Study 1	Weisman et al. (2017, PNAS)	Weisman et al. (2017, Proc. CogSci)	Gray et al. (2007)	Malle (2019, Study 1)	Malle (2019, Study 2)
feeling embarrassed	feeling embarrassed	feel embarrassed	embarrassment	[similar to ‘can feel shame or pride’]	-
feeling pride	experiencing pride	feel proud	pride	can feel shame or pride	-
making choices	making choices	make choices	-	can choose freely	-
calming themselves down	-	-	-	-	-
detecting danger	-	-	-	-	-
feeling hopeless	-	-	-	-	-
remembering things	remembering things	remember things	memory	can remember things	-
imagining things	-	-	-	can vividly imagine things	vividly imagining things
recognizing others’ emotions	understanding how others are feeling	understand how somebody else is feeling	emotion recognition	[similar to ‘can have empathy for others’]	[similar to ‘feeling compassion’]
feeling worried	-	-	-	-	-
getting hurt feelings	[similar to ‘feeling disrespected’]	get hurt feelings	-	-	-
having wants and desires	having desires	have desires	desire	can want certain things	having desires
**Not included**					
-	being conscious	-	consciousness	-	-
-	being self-aware	be aware of itself	-	-	-
-	communicating with others	communicate with somebody else	communication	can communicate with others	communicating verbally
-	detecting odors	-	-	-	-
-	doing computations	-	-	-	-
-	exercising self-restraint	-	-	-	-
-	experiencing joy	feel joy	joy	can feel joy	-
-	feeling depressed	-	-	-	-
-	feeling disrespected	-	-	-	-
-	feeling nauseated	-	-	-	-
-	having a personality	have a personality	personality	-	-
-	having free will	-	-	-	-
-	having intentions	-	-	-	-
-	holding beliefs	-	-	can believe certain things	-
-	perceiving depth	-	-	-	-

Table 23: Item-by-item comparison of the capacities included in the current studies to capacities included in previous studies of mind perception. *(continued)*

Study 1	Weisman et al. (2017, PNAS)	Weisman et al. (2017, Proc. CogSci)	Gray et al. (2007)	Malle (2019, Study 1)	Malle (2019, Study 2)
-	sensing temperatures	sense temperatures	-	-	-
-	-	decide what to do	-	-	-
-	-	do math	-	-	-
-	-	feel sick	-	-	-
-	-	figure out how to do things	-	-	-
-	-	have beliefs	-	-	-
-	-	know what's nice and what's mean	-	-	-
-	-	make plans	-	-	-
-	-	sense whether someting is close by or far away	-	-	-
-	-	smell things	-	can taste or smell things	can taste or smell things
-	-	-	-	can have empathy for others	-
-	-	-	-	can have values	upholding moral values
-	-	-	-	can imitate others	-
-	-	-	-	can know certain things	-
-	-	-	-	can perceive things	-
-	-	-	-	has moral obligations	-
-	-	-	-	may deserve praise or blame	-
-	-	-	-	may deserve punishment	-
-	-	-	-	-	communicating non-verbally
-	-	-	-	-	disapproving of immoral actions
-	-	-	-	-	disliking people
-	-	-	-	-	explaining their decisions to others
-	-	-	-	-	feeling compassion
-	-	-	-	-	feeling gratitude
-	-	-	-	-	feeling panic

Table 23: Item-by-item comparison of the capacities included in the current studies to capacities included in previous studies of mind perception. *(continued)*

Study 1	Weisman et al. (2017, PNAS)	Weisman et al. (2017, Proc. CogSci)	Gray et al. (2007)	Malle (2019, Study 1)	Malle (2019, Study 2)
-	-	-	-	-	feeling sexual arousal
-	-	-	-	-	feeling stress
-	-	-	-	-	feeling temperature, touch, etc.
-	-	-	-	-	following norms
-	-	-	-	-	having emotions
-	-	-	-	-	having intense urges
-	-	-	-	-	inferring what a person is thinking
-	-	-	-	-	learning by imitation
-	-	-	-	-	moving on their own
-	-	-	-	-	praising moral actions
-	-	-	-	-	providing reasons for their actions
-	-	-	-	-	taking a person's visual point of view
-	-	-	-	-	understanding a person's goals

Notes: * Also used in Study 2; † Also used in Study 3

References

American Academy of Pediatrics. (2018). *AAP schedule of well-child care visits.* https://www.healthychildren.org/English/family-life/health-management/Pages/Well-Child-Care-A-Check-Up-for-Success.aspx

Bates, D., Maechler, M., Bolker, B., & Walker, S. (2015). lme4: Linear mixed-effects models using eigen and S4. *Journal of Statistical Software*, *67*(1), 1–48. https://doi.org/10.18637/jss.v067.i01

Centers for Disease Control and Prevention. (2021). *CDC's developmental milestones.* https://www.cdc.gov/ncbddd/actearly/milestones/index.html

Gray, H. M., Gray, K., & Wegner, D. M. (2007). Dimensions of mind perception. *Science*, *315*(5812), 619. https://doi.org/10.1126/science.1134475

Malle, B. F. (2019). How many dimensions of mind perception really are there. In A. K. Goel, C. M. Seifert, & C. Freksa (Eds.), *Proceedings of the 41st annual meeting of the cognitive science society* (pp. 2268–2274).

Owen, A. B., & Wang, J. (2016). Bi-cross-validation for factor analysis. *Statistical Science*, *31*, 119–139. https://doi.org/10.1214/15-STS539

Putnam, S. P., Gartstein, M. A., & Rothbart, M. K. (2006). Measurement of fine grained aspect of toddler temperament: The early childhood behavior questionnaire. *Infant Behavior and Development*, *29*(3), 386–401. https://doi.org/10.1016/j.infbeh.2006.01.004

Revelle, W. (2021). *Psych: Procedures for psychological, psychometric, and personality research.* Northwestern University. https://CRAN.R-project.org/package=psych

Rothbart, M. K. (1978). *Infant behaviour questionnaire (IBQ).*

Venables, W. N., & Ripley, B. D. (2002). *Modern applied statistics with S* (Fourth). Springer. https://www.stats.ox.ac.uk/pub/MASS4/

Weisman, K., Dweck, C. S., & Markman, E. M. (2017a). *Children's intuitions about the structure of mental life.* 1333–1338.

Weisman, K., Dweck, C. S., & Markman, E. M. (2017b). Rethinking people's conceptions of mental life. *Proceedings of the National Academy of Sciences*, *114*(43), 11374–11379. https://doi.org/10.1073/pnas.1704347114

Weisman, K., Dweck, C. S., & Markman, E. M. (2018). *Folk philosophy of mind: Changes in conceptual structure between 4-9y of age.* 1163–1168.

Weisman, K., Legare, C. H., Smith, R. E., Dzokoto, V. A., Aulino, F., Ng, E. K., Dulin, J. C., Ross-Zehnder, N., Brahinsky, J. D., & Luhrmann, T. M. (2021). Similarities and differences in concepts of mental life among adults and children in five cultures. *Nature Human Behavior.*

Wood, S. N. (2017). *Generalized additive models: An introduction with r* (Second). Chapman; Hall/CRC.

Xie, Y. (2016). *Bookdown: Authoring books and technical documents with R markdown.* Chapman; Hall/CRC. https://bookdown.org/yihui/bookdown

Xie, Y. (2021). *Knitr: A general-purpose package for dynamic report generation in r.* https://yihui.org/knitr/

Xie, Y. (2024). *Bookdown: Authoring books and technical documents with r markdown.* https://github.com/rstudio/bookdown
